# Formation of helical membrane tubes around microtubules by single-headed kinesin KIF1A

**DOI:** 10.1038/ncomms9025

**Published:** 2015-08-13

**Authors:** David Oriola, Sophie Roth, Marileen Dogterom, Jaume Casademunt

**Affiliations:** 1 Departament d'Estructura i Constituents de la Matèria, Facultat de Física, Universitat de Barcelona, Avinguda Diagonal 647, E-08028 Barcelona, Spain; 2 FOM Institute AMOLF, Science Park 104, 1098 XG Amsterdam, The Netherlands

## Abstract

The kinesin-3 motor KIF1A is in charge of vesicular transport in neuronal axons. Its single-headed form is known to be very inefficient due to the presence of a diffusive state in the mechanochemical cycle. However, recent theoretical studies have suggested that these motors could largely enhance force generation by working in teams. Here we test this prediction by challenging single-headed KIF1A to extract membrane tubes from giant vesicles along microtubule filaments in a minimal *in vitro* system. Remarkably, not only KIF1A motors are able to extract tubes but they feature a novel phenomenon: tubes are wound around microtubules forming tubular helices. This finding reveals an unforeseen combination of cooperative force generation and self-organized manoeuvreing capability, suggesting that the diffusive state may be a key ingredient for collective motor performance under demanding traffic conditions. Hence, we conclude that KIF1A is a genuinely cooperative motor, possibly explaining its specificity to axonal trafficking.

## Abstract (web summary)

The kinesin-3 motor KIF1A is inefficient in its single-headed form but recent studies have predicted that force transmission is enabled when motors work in teams. Here Oriola *et al.* show that singleheaded KIF1A motors can pull tubes from lipid vesicles, and form left-handed helices around microtubules by generating lateral forces.

## Graphical abstract

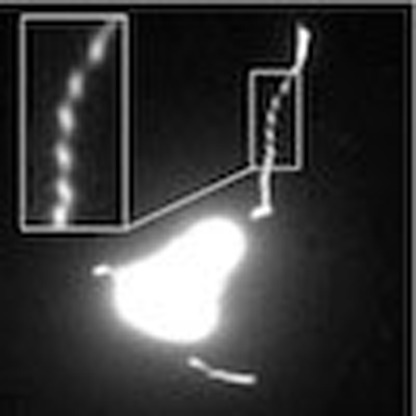

Intracellular trafficking is essential for the maintenance of cell functions. Plus- and minus-end directed molecular motors move along microtubule (MT) filaments, transporting organelles to specific locations in cells^1^,^2^. The ability of cargo-bound motors to manoeuvre through obstructions and overcome possible roadblocks is crucial to ensure efficient transport. Recently, superresolution microscopy has revealed how motors manage to squeeze membrane-bound cargos, such as lysosomes, through narrow gaps^3^. In axonal transport, the capability to circumvent obstacles or overcome obstructions by exerting large forces becomes even a more pressing issue, since cargo delivery must be secured over extremely long distances in a particularly crowded and constrained environment^4^,^5^,^6^. Specifically, traffic dysfunctions in axonal transport, for instance in the form of traffic jams, have been associated to several neurodegenerative diseases^7^.

KIF1A is a plus-end kinesin-3 family motor specific of axonal transport of synaptic vesicle precursors^5^,^8^. This motor has been implicated in neuronal disorders^6^,^9^ and viral trafficking^10^. Both its monomeric (single-headed) and dimeric (two-headed) forms have been studied extensively^8^,^11^,^12^,^13^,^14^,^15^,^16^. In both the cases these motors have been shown to combine a strongly bound state to the MT with a weakly bound state that allows them to diffuse without completely detaching from the MT^13^,^15^. A truncated single-headed form of KIF1A was proposed by Hirokawa and colleagues^13^ as a model motor, well described by a two-state noise-driven ratchet mechanism^17^,^18^,^19^,^20^. Single-molecule experiments with single-headed KIF1A reported velocities of 0.2 μm s^−1^ and very small stall forces around 0.1 pN (ref. 13). On the other hand, KIF1A has been found to be dimeric *in vivo* eliciting large velocities (∼1 μm s^−1^), alternating a diffusive state and a processive state^15^. Dimerization of KIF1A has been reported to be mainly regulated through the neck coil segment, surprisingly resulting into superprocessive motion^16^. Still, the diffusive state is at odds with the demanding conditions of axonal transport since it makes the individual motor inefficient and weak compared with conventional dimeric kinesin (kinesin-1), which has a stall force of ≃6 pN and moves processively using a hand-over-hand mechanism^2^,^21^. Recently, however, theoretical studies have shown that interacting motors can cooperate in groups^22^,^23^. In particular, the existence of a diffusive state may be an important advantage to allow force transmission and cooperation when motors team up in membrane-bound cargoes^24^,^25^, specially in the case of KIF1A (refs 26, 27). Interestingly, this cumulative effect seems to be missed by conventional kinesin^28^,^29^. In addition, the diffusive state could favour the transversal motion of motors to change protofilaments and enhance manoeuvrability.

As a first step to understand the specificity of KIF1A in axonal transport and the existence of a diffusive state, we study the most unfavourable and simplest case, namely its single-headed form. Our aim is to prove the hypothesis that force transmission is enabled by the diffusive state when multiple motors work in teams. We test experimentally the theoretical scenario predicted in ref. 26, by challenging single-headed KIF1A motors to extract membrane tubes from giant unilamellar vesicles (GUVs). This minimal *in vitro* setup has been widely used to probe the collective action of membrane-bound molecular motors^30^,^31^,^32^,^33^,^34^,^35^, and it was originally conceived to mimic the formation of membrane tube networks *in vivo*^36^. Although KIF1A has not been implicated in tube formation, the closely related kinesin-3 forms, KIF13A and KIF16B, have been reported to induce tubulation *in vivo*^37^,^38^. Additionally, recent results from ref. 35 have specifically suggested the interest to test KIF1A collective force generation using tube-pulling assays. Here, we confirm that single-headed KIF1A motors are indeed capable to extract tubes in similar conditions to conventional kinesin, despite having a stall force 60 times smaller. This surprising fact proves the unique adaptation of single-headed KIF1A motors to cooperative force generation. A microscopic *in silico* model is developed showing how motors team up and cooperate to enable tube extraction. In addition, we find that the motors exhibit a systematic left-handed transversal bias that twists the tubes to form regular helices winding around MTs, with a certain pitch variability. While left-handed chirality has been reported for several kinesin motors moving along MTs^39^,^40^,^41^, this is the first time that molecular motors are shown to collectively generate significant lateral forces to the point of producing the spiralling of membrane tubes. Remarkably, this is achieved even though the motor has an extremely small individual off-axis stall force, estimated to be ≃0.04 pN from our experiments. Finally, we describe helical tube formation by using a mean-field model which accounts for the dynamical selection of the pitch by the collective action of motors, as a consequence of the self-organized structure of the motor cluster at the tube tip.

## Results

### Single-headed KIF1A induces parallel and helical tubulation

**Figure 1:**
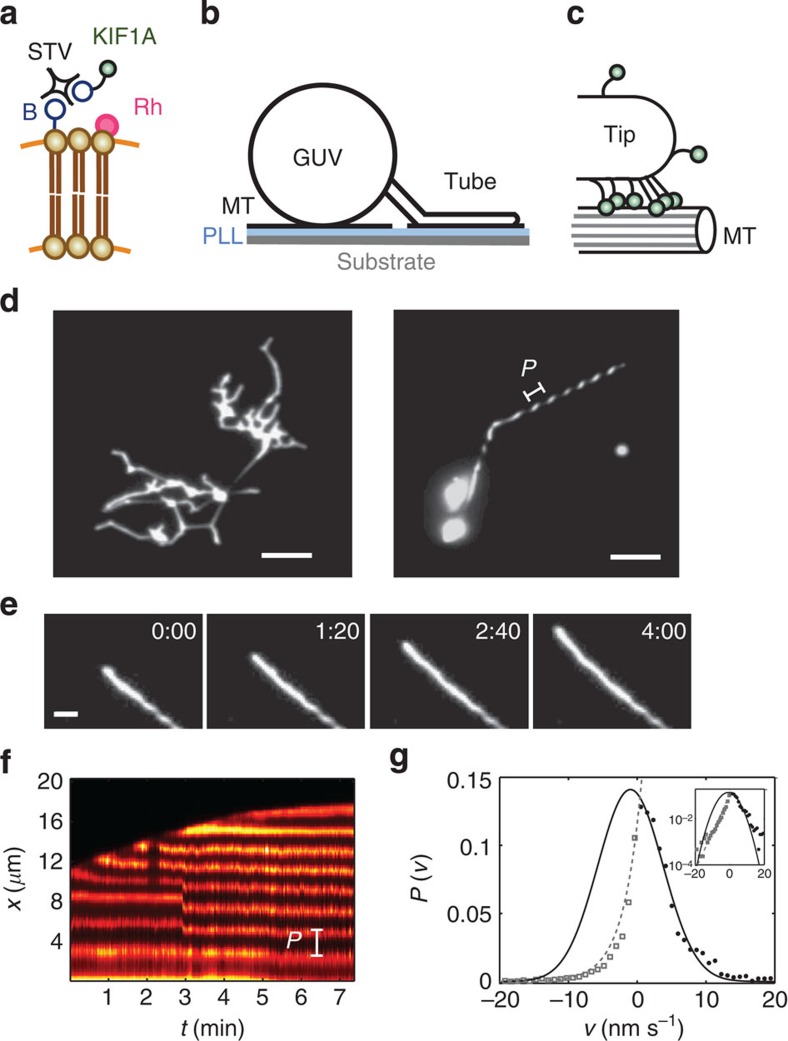
Experimental setup and dynamics of tube extraction. (**a**) Biotin (B)–streptavidin (STV) linkage of KIF1A with the GUV bilayer (yellow). The lipid mixture contains a small fraction of rhodamine (Rh)-labelled phospholipids. (**b**) Schematic description of the experimental setup: poly-L-Lysine (PLL) is used to attach MT to the substrate and GUVs are sedimented on top. Tubes grow upon the addition of ATP in the system. (**c**) Tip region where KIF1A motors accumulate. (**d**) TIRF fluorescent images of membrane tubes: (left) membrane tube network formed on the underlying MT network. (right) Helical tube extracted from a GUV. *P* is a measure of the tube pitch and is defined as the peak-to-peak distance between intensity maxima along the tube. Scale bars, 5 μm. (**e**) Time lapse of a tube growing in minutes. Scale bar, 2 μm. (**f**) Kymograph of the growing helical tube in **d** right (Supplementary Movie). Notice that around *t*=3 min a rapid relaxation of the pitch is observed. (**g**) Instantaneous velocity distribution *P*(*v*) using 24 events of longitudinal pulling from 11 replicates. The distribution is clearly asymmetric and can be interpreted by considering retraction and growth as two differentiated processes. Lines show the best fit to the distribution for negative (□) and positive (●) velocities using exponential (dashed line) and gaussian (solid line) profiles respectively. Inset: Same distribution in the logarithmic scale.

We studied the collective action of single-headed KIF1A by linking the biotinylated motor proteins via streptavidin to a fraction of biotinylated lipids on the GUVs' surface (Fig. 1a). The conditions used were similar to previous experiments with conventional kinesin^32^. In our experiments, we used the previously studied truncated form of KIF1A (1–382 amino acids) reported to be monomeric^13^. We found that, upon sedimentation of the GUVs on a MT-coated surface in the presence of ATP, KIF1A motors were able to extract membrane tubes (Fig. 1b,c) and networks were formed (see Fig. 1d, left). Different fractions of biotinylated lipids were studied, ranging from 0.01 to 1 mol%. For the case of 1 mol%, networks of tubes were formed in minutes whereas for 0.01–0.1 mol%, few tubes were formed after more than an hour. The 0.01 mol% case was found to be close to the threshold motor surface density for tube formation. This threshold value is comparable to the one for conventional kinesin^32^. The sole fact that tubes are being extracted despite the inherent weakness of individual KIF1A motors is by itself a proof of the existence of a strong cooperative effect such as that predicted in refs 26, 27, even though the precise mechanism cannot be inferred from the experiment.

Tube growth velocities ranged from 2 to 20 nm s^−1^ (much smaller than MT gliding velocities, see Methods), indicating that motors worked near stall conditions at the tip. Moreover, velocities did not vary significantly in the range 0.1–1 mol%, in contrast to tube-pulling experiments with myosin^35^. This is consistent with the prediction that the collective velocity–force relationship saturates with the number of motors^27^. Hence, in our case, the tip velocity becomes very weakly dependent on the density of motors at the GUV.

In three cases we observed episodes of slow backward motion, with characteristic velocities of ∼4 nm s^−1^ indicating the presence of bidirectional movement. Similar slow backward movements were reported in the case of non-processive Ncd motors^33^, due to the presence of motors distributed all along the tube, typically forming motor clusters capable to withstand tube retractions. In that case, the clustering mechanism resulted from the diffusive motion of motors on the MT lattice due to their non-processivity^34^. Our case seems to obey a similar scenario, however diffusion along the MT is now associated to the inherent diffusive state of KIF1A. We measured instantaneous speeds for individual tip traces by subtracting end point positions of a window moving along the trace (see Methods). In Fig. 1g the instantaneous velocity distribution of the tube tip is shown. The distribution is clearly asymmetric. This fact can be understood by considering shrinkage and growth as two distinct processes. Following ref. 33, the distribution for negative displacements may be explained by assuming a random cluster distribution due to the diffusive nature of KIF1A, which leads to an exponential distribution of retraction distances. For positive displacements, instead, the observed statistics reflects the interplay between a ballistic component and the diffusive spread, which suggests a Gaussian distribution. This asymmetry is validated by our simulations of the system (see *in silico* model for longitudinal tube pulling).

**Figure 2:**
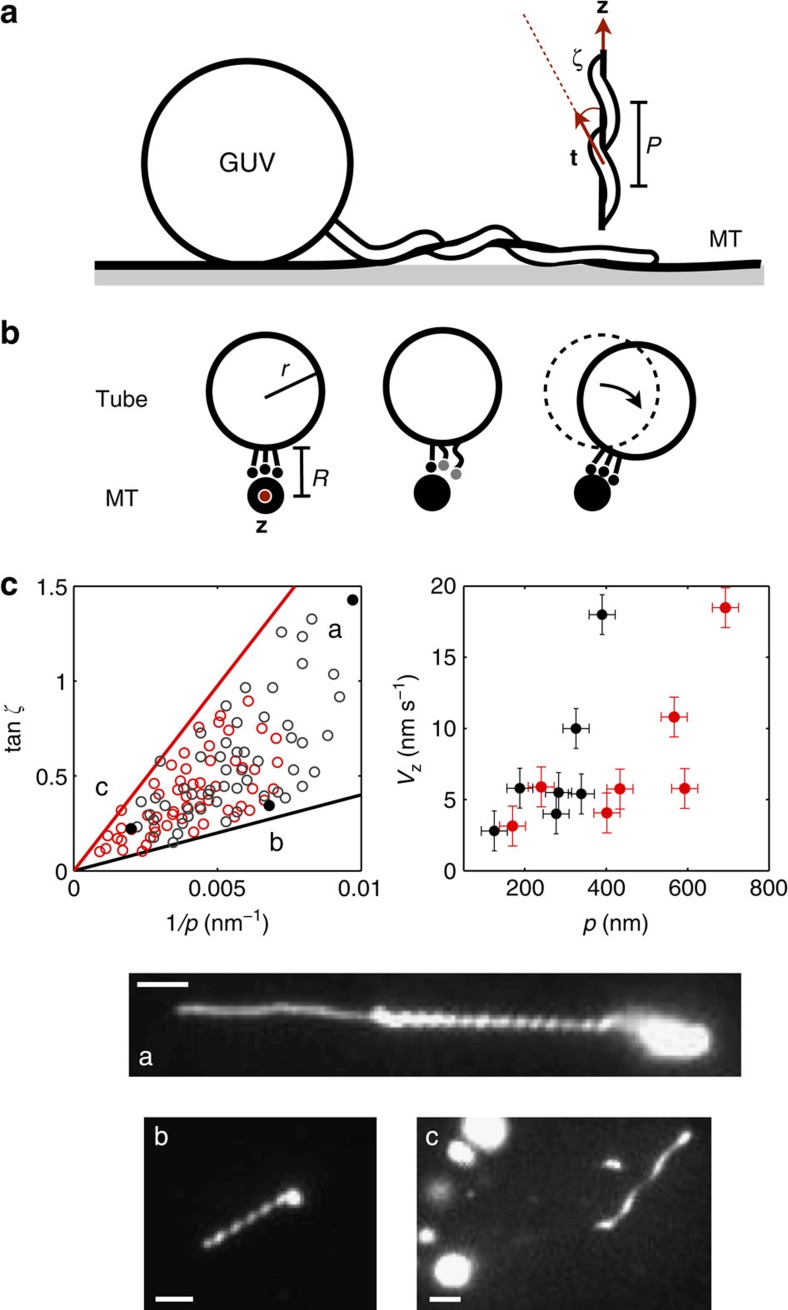
Geometry and analysis of the helical tubes. (**a**) Schematic description of the formation of a helical tube from a GUV around a MT. Some defects in the MT network allow motors to pull tubes through nanometre range gaps between the MT and the substrate. The angle *ζ* and the pitch *P* characterize the geometry of the helix. (**b**) Off-axis movement of the tube: (left) motors are found initially in a strongly bound conformation (black). (Centre) Some of them switch to the weakly bound state (grey) and progressively switch protofilaments by diffusion. (Right) When motors return to the strongly bound state, the tube turns counter clockwise. (**c**) (Top, left) tan *ζ* versus 1/*p* plot using 57 helical tubes from 11 replicates (grey circles) and the shifted data by assuming all MTs have 14 protofilaments (red circles) with a superpitch of 6 μm. The two lines show the lower limit *R*_0_=40 nm (black line) and the upper limit *R*_0_=195 nm (red line). The measurement error is ±10^−3^ nm^−1^ in the horizontal axis and ±0.1 in the vertical axis. (Top, right) On-axis tip velocity versus the angular pitch. Error bars correspond to s.d. (vertical) and measurement error (horizontal). (Bottom) TIRF images of helical tubes corresponding to the black filled circles (a—c) in **c** (top, left). Scale bars, 2 μm.

The second main result is that approximately half of the extracted tubes wound around the MTs forming left-handed helical structures with well-defined pitch (Fig. 1d, right). In some cases membrane tube networks exhibited mixed longitudinal and helical tube formation. In the case of growing helical tubes, MTs usually fluctuated close to the focal plane, indicating that they were partially anchored to the substrate and consequently tubes were able to grow underneath MTs (Supplementary Movie and Fig. 2a). We attribute the longitudinal growth to the cases in which MTs were strongly attached to the substrate. Plausibly, the ability of KIF1A motors to switch protofilaments is facilitated by the existence of the weakly bound state (Fig. 2b), similarly to the case of single-headed kinesin-1 (ref. 40). Hence, this state provides a certain freedom for the motor to switch between on-axis or off-axis movements, a relevant feature when the motor runs into obstacles^42^. The leftward bias observed in some kinesin motors is thought to be originated in the intrinsic left–right asymmetry of the motor–MT interaction^40^,^43^, which in turn reflects MT chirality. However, it is not obvious that such an intrinsic bias is sufficient to collectively generate significant off-axis forces up to the point of twisting the membrane tubes in a counter clockwise motion around the MT. In Fig. 1d (right) the pitch *P* is measured as the distance between two consecutive fluorescence maxima. The helical pitch is observed to be regular along the helix although sometimes the helical turns rearrange dynamically via slow (minutes) or rapid (seconds) rearrangements (Fig. 1f) converging to a homogeneous pitch. The average value of the pitch was found to be 1.4±0.1 μm (see Methods). Next, we study the geometry of the helical tubes. We define the pitch *P* as the length of MT covered per turn of the helix, and the angular pitch as *p* ≡ *P*/2*π*. We define *ζ* as the angle the tangent vector of the tube axis **t** forms with respect to the MT axis **z** (Fig. 2a). From the geometry of a helix (Supplementary Note 1) we have tan *ζ*=*R*_0_/*p*, where *R*_0_ ≡ *r*+*R*, *r* is the radius of the tube and *R* is the radius of the MT plus the extra space occupied by the motors (Fig. 2b and Supplementary Figure 1).

The tube radius *r* results from the balance of bending energy and surface tension. For a straight tube, energy minimization yields the minimum pulling force to extract a tube 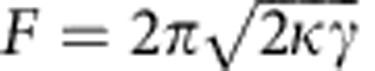, and the tube diameter 
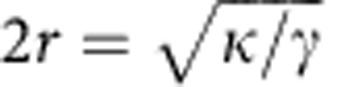
, where *κ* is the bending modulus of the membrane and *γ* the surface tension^44^. For a helical tube, the twist will introduce additional bending energy due to mechanical work performed by the motors. Typically the values of both *F* and *r* from the straight case are reasonably accurate for the actual helical tubes (Supplementary Note 1), an exception being the point (a) in Fig. 2c. The selection of the pitch is thus a dynamic process that results from the collective action of the motors. Once the tube is formed; however, the winding number is conserved as a topological constraint imposed by the presence of the MT, and energy minimization will only tend to leave a uniform pitch. Hence, the observed pitch inhomogeneities in Fig. 1f are a consequence of the motor activity.

In Fig. 2c (top, left) we show experimental data points of tubes forming left-handed helices with pitch *P* and angle *ζ* (grey circles). *In vivo* MTs typically contain 13 protofilaments, which run straight with respect to the MT axis. However, MTs grown *in vitro* may contain a similar fraction of 14 protofilament MT^45^,^46^. In the latter case, protofilaments wind around the MT axis, and introduce an extra pitch (superhelical pitch) in the helical tubes. In order to account for this effect, the red circles in Fig. 2c correspond to exactly the same data but subtracting the possible extra pitch introduced by 14 protofilament MTs (Supplementary Note 1). This correction is small provided that the pitch of the helix is much smaller than the superhelical pitch. The cloud of points falls into a certain sector of the parameter space bounded by a black line (*R*_0_=40 nm) and a red line (*R*_0_=195 nm). The scattering of points in Fig. 2c (top, left) reflects the variability of surface tension from vesicle to vesicle, which in turn yields a variety of tube radii. Tubes (a) and (c) in Fig. 2c have completely different pitches despite having similar *R*_0_, implying different tip velocities. In Fig. 2c (top, right), we see that the *z*-component of the tip velocity *V*_*z*_ grows as a function of *p*, suggesting that the shape of the helix roughly follows the trace of the tube tip during growth. Therefore, we conclude that the pitch grows for increasing tip velocity.

Note that from the measurement of *p* and *ζ*, the helix geometry provides a simple way to measure the tube radius *r* and consequently the membrane tension *γ*, which is usually subject to larger uncertainty than the bending rigidity, provided that the distance between the MT and the tube is known. A simple estimation based on a size ∼5 nm for the biotin–streptavidin–biotin complex, a motor domain of KIF1A of ∼6 nm (ref. 11), and a contour length of the construct neck linker of ≈8 nm yields *R*≃12–30 nm and thus a tube radii variability of *r*≃10–180 nm. Assuming *κ*=10*k*_B_*T* we estimate the membrane tension to be in the range *γ*≃3 × 10^−4^–10^−1^ pN nm^−1^ and *F*≃1–20 pN.

### *In silico* model for longitudinal tube pulling

To understand at a quantitative level the on-axis cooperative force generation of single-headed KIF1A, we first describe tube pulling for the case of firmly attached MTs, in which tubes grow longitudinally along the MT axis. This problem has been studied theoretically for conventional kinesin using mean-field and lattice approaches^32^,^47^. Here we will use a Brownian dynamics approach, extending the previous work of refs 26, 27 in order to include the attachment/detachment kinetics of the motors between the tube and the MT and to mimic the conditions of the *in vitro* system. This method explicitly allows force transmission between motors via exclusion volume interactions, which is the key ingredient for cooperativity in the system.

**Figure 3:**
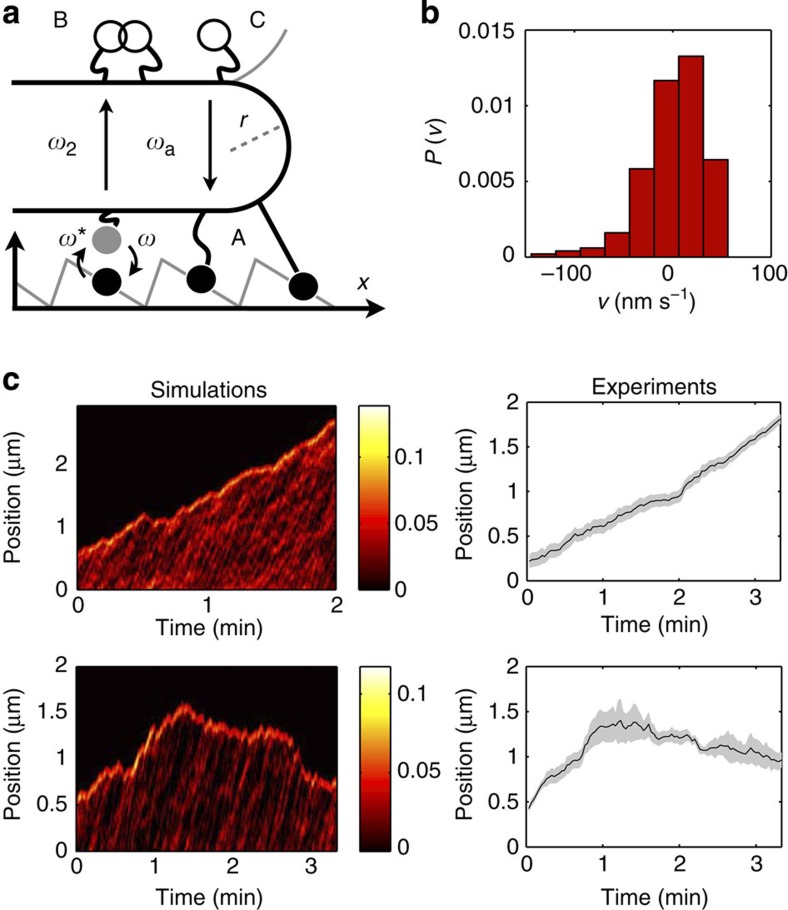
*In silico* model for longitudinal tube pulling. (**a**) Schematic description of the tube tip. Three different regions can be identified (A–C). In region A, motors are either strongly bound (black circles) or weakly bound (grey circles) to the MT. They are excited and decay with average rates ω*, *ω* respectively. In region B, motors are detached from the MT and diffuse freely away from the tip, where overlapping is allowed. Motors switch between regions A and B with mean rates *ω*_2_ and *ω*_a_ via detachment or attachment transitions respectively. Finally, detached motors in region C feel a soft repulsion which prevents them to enter the tip region (Supplementary Note 2). (**b**) Instantaneous tip velocity distribution using the data in **c** (bottom, left). For illustrative purposes, the time window was chosen 1 s to improve the statistics at the expense of increasing the dispersion. (**c**) Growth (top) and bistable motion (bottom) of a membrane tube. (Left) Simulation of the motor density plot showing tube growth with *ρ*_∞_=1,000 μm^−2^ (top) and *ρ*_∞_=200 μm^−2^ (bottom), *γ*=0.05 pN nm^−1^, *κ*=10*k*_B_*T*. The rest of parameters are specified in the Supplementary Table 1. The colour bar indicates the density of bound motors (arbitrary units). (Right) Experimental trajectories of the tube tip for 0.1 mol% biotinylated lipids. The grey region depicts the uncertainty of the tip position corresponding to 1.96 s.d. (see Methods). We find excellent quantitative agreement between velocities in experiments and in simulations.

We model the problem as a one-dimensional arrangement of interacting KIF1A motors, each one described by a two-state noise-driven ratchet to account for the strongly/weakly bound states to the MT, and attachment/detachment kinetics between the MT and the tube (Fig. 3a). The number of motors bound to the tube is a fluctuating quantity set by the influx of motors in the vesicle–tube junction, which is mainly controlled by the surface density of motors *ρ*_∞_ on the GUV (Supplementary Fig. 2). We assume that the arrangement of motors at the tip is such as depicted in Fig. 2b (left) where they occupy three different protofilament tracks, similarly to the case of conventional kinesin, as discussed in ref. 47. For simplicity we neglect interactions between motors in neighbouring protofilaments and assume that the on-axis cooperativity can be reduced to a single-protofilament problem, scaling down the total force *F* and the motor density *ρ*_∞_ by a factor 3. In the tube region, a given motor can be in three possible states: detached from the MT, strongly bound to the MT or weakly bound to the MT (white, black and grey circles respectively in Fig. 3a) and its dynamics will be different depending on the region where it is found (A, B or C). Motors are excited and decay with rates ω*, *ω* and attach/detach to the MT with rates *ω*_a_ and *ω*_2_ respectively. A detailed description of the model can be found in the Supplementary Note 2.

In Fig. 3c (left), the dynamics of the tube tip and the motor density over time are shown. Motors work collectively at the tube tip against the external load by means of a cooperative mechanism previously reported in ref. 26 for the case of no attachment/detachment kinetics. Here, the exchange kinetics controls the size of the tip cluster, together with other parameters such as the potential height *U* (Supplementary Note 2), ω* and *γ*. Although many motors are involved in the process, the tube can be extracted provided that an average number of motors *n*_c_ are packed at the tip sharing the load. For typical values *U*=10–20*k*_B_*T*, *γ*≃0.1 pN nm^−1^ and ω* on the order of hundreds of Hz, we have *n*_c_≃12, only slightly larger than the typical values of 6–9 estimated for experiments with conventional kinesin in ref. 47. We notice that motors not only accumulate at the tube tip but they are also present with significant density all along the tube. In Fig. 3c (top, left), we show the tube growth for *ρ*_∞_=1,000 μm^−2^ and *γ*=0.05 pN nm^−1^. The total force is ∼12 pN, the average number of bound motors in the tip cluster is ∼15 and the tube grows with a roughly constant velocity ∼15 nm s^−1^. These values are in quantitative agreement with the experimental observed velocities (Fig. 3c, right, top) and the indirectly inferred extraction forces. In this case, the number of motors in the tip fluctuates with an average value which is above the threshold value for tube extraction. However, in Fig. 3c (bottom, left), the influx of motors is reduced (*ρ*_∞_=200 μm^−2^), and the average number of motors is close to *n*_c_. Although the surface density of motors is decreased fivefold, the velocity during the growth phase in Fig. 3c (left, bottom) is similar to the *ρ*_∞_=1,000 μm^−2^ case, in agreement with the weak dependence of the velocity on the surface density of motors in the experiments. We observe rapid and slow retractions which are rescued by motor density waves advancing along the tube, with an overall retraction velocity of 4 nm s^−1^. The same bistable motion is found experimentally (Fig. 3c, right, bottom). In Fig. 3b, the instantaneous tip velocity distribution is shown for the data in Fig. 3c (left, bottom). We observe that the distribution is in qualitative agreement with the experimental results in Fig. 1g, capturing the asymmetry of the distribution. Hence, our *in silico* model reproduces both the growth and the bistable motion of tubes.

### Mean-field model for helical tube formation

Finally, we introduce a mean-field model to understand the role of off-axis forces in helical tube formation when MTs are not firmly attached to the surface. Our goal is to understand the dynamical selection of the helical pitch from the collective arrangement of motors in the MT lattice. First, we need to describe how single-headed KIF1A motors are able to change protofilament tracks along the MT. This problem was first studied in the context of traffic flow on a lattice for the case of no load applied to the motors^48^. Here we will use a similar approach based on an extension of the work in ref. 26.

We consider the MT surface as an oblique Bravais lattice with primitive vectors **r**_1_, **r**_2_ forming an angle *θ*, with lenghts *l*_1_, *l*_2_ which correspond to the tubulin heterodimer distance along a protofilament and the nearest tubulin heterodimer distance between adjacent protofilament, respectively (Supplementary Fig. 3). Each node on the lattice corresponds to a binding site for the motor. We consider the motion of KIF1A to be a superposition of on-axis and off-axis movements, following the primitive directions. These movements are considered as independent processes, which has been shown to be a reasonable assumption for single-headed kinesin-1 (ref. 40). We assume motors can change protofilament tracks only when they diffuse in the weakly bound state. In order to account for the extra biasing process observed in experiments, we need to incorporate a second asymmetry parameter describing the motor–MT landscape potential. Taking into account the previous ingredients in a lattice model for KIF1A, one can show that linear velocity–force relationships are recovered in each primitive direction *i*, with velocities at zero load *v*_*i*_(0) and stall forces 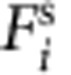 which depend on microscopic parameters (Supplementary Note 3). Defining the ratio 
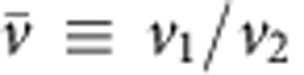
, the average angle of a single-motor helical trajectory *ζ*_1_ can be found as a function of 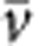 and *θ* through the expression 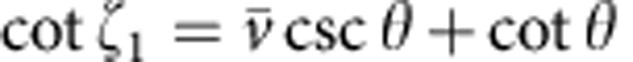. The average pitch of a helical trajectory around the MT will be given by *P*_1_=2*πR*_MT_ cot *ζ*_1_ where *R*_MT_ is the MT radius. Notice that approximating *θ*≃*π*/2, we have 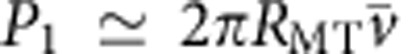 and the pitch is proportional to 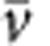. At zero load we obtain a simple expression for the single-motor average pitch *P*_1_(0) ≃2*πR*_MT_ (*l*_1_−2*a*_1_)/(*l*_2_−2*a*_2_). Considering the typical values for a MT lattice, we have *l*_1_≃8 nm, *l*_2_≃6 nm and *θ*=81° (ref. 49). Assuming zero load, *R*_MT_≃12 nm and *a*_1_∼*a*_2_ we get *P*_1_∼100 nm, which coincides with the order of magnitude of the reported pitch for single-headed kinesin-1 (ref. 40), a motor relatively similar to KIF1A. In order to estimate 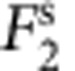, we adjust the asymmetries *a*_1_ and *a*_2_ to match the experimental pitch of single-headed kinesin-1 (≃300 nm), thus obtaining 
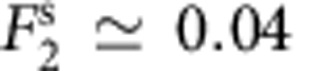
 pN for *a*_1_/*l*_1_=0.2 and *a*_2_/*l*_2_=0.4.

**Figure 4:**
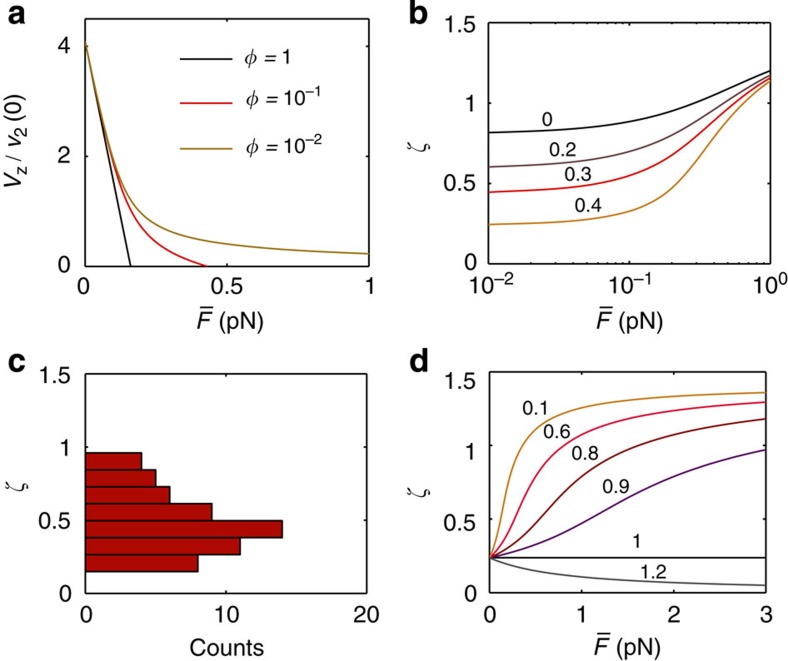
Mean-field description of helical tube formation. (**a**) Dimensionless on-axis velocity of the tube respect to 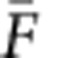 for different *φ* and *a*_2_/*l*_2_=0.4. We notice the apparition of long tails in the velocity–force relationship for decreasing *φ*. (**b**) Angle dependence on the force per motor 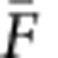 for different *a*_2_/*l*_2_ values and *φ*=0.5. (**c**) Experimental angle distribution of 57 standing helical nanotubes. (**d**) Angle dependence on the force per motor 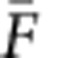 for different *φ* values and *a*_2_/*l*_2_=0.4. *θ*=81°, 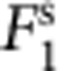
=0.15 pN and *a*_1_/*l*_1_=0.2. Angles are shown in radians.

Next we study the process of pitch selection during the formation of a tubular helix by the collective action of the motors. The pitch of the tube will result from the competition of the total on-axis and off-axis forces, a nontrivial combination of two collective effects that depend on the actual distribution of motors at the tip cluster and the different mechanisms of cooperation for serial and parallel arrangements of motors. In contrast to the single-motor case, if the applied force is exerted by the membrane, the force components *F*_*i*_ are dependent on *ζ* and *θ* through *F*_*i*_=*Fg*_*i*_(*θ*, *ζ*), where *g*_1_(*θ*, *ζ*)=cos *ζ*−sin *ζ* cot *θ*, *g*_2_(*θ*, *ζ*)=sin *ζ* / sin *θ* and *F* is the extraction force. The balance between the membrane tension and the collective motor forces along the two directions of the lattice will eventually select a mean orientation angle in the range [0, *θ*]. For simplicity, we assume that each component of the total force is equally shared by a certain group of motors. Therefore, *V*_*i*_(*F*_*i*_)=*v*_*i*_(*F*_*i*_/*N*_*i*_), where *N*_*i*_ is the number of motors generating force along the *i*-th direction. Defining 
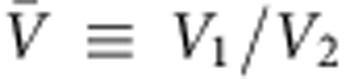
 we have:


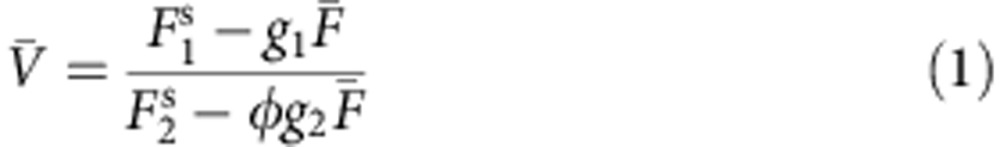


where 
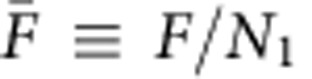
 is the effective force per motor and *φ* ≡ *N*_1_/*N*_2_. On the other hand, we know that 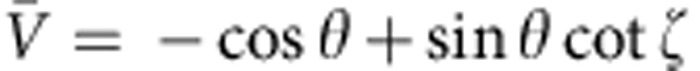. By equating the last expression and equation (1), we obtain a transcendental equation for *ζ* which can be solved numerically. The dynamics of a helical tube and the angle selection are crucially affected by the phenomenological parameter *φ*. In Fig. 4a, for *φ*=1, the on-axis velocity–force relationship is linear and *ζ* is independent of the extraction force *F*. However, for *φ*<1, long tails appear on the on-axis velocity–force relationship and *ζ* becomes strongly dependent on *F* (Fig. 4b,d). In the latter case, the on-axis velocity may decrease by a factor four under moderately large forces, consistently with our tube-pulling data in comparison with gliding assays (see Methods). In Fig. 4c the experimental angle distribution is shown by taking the average angle of 57 helical tubes. We compare the data with the dependence of *ζ* on 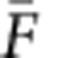 for different values of *φ* (Fig. 4d). Considering 
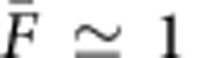
 in the experiments, the range *φ*≃0.6–1.2 approximately bounds the experimental angle values. We can also infer the total off-axis force exerted by the motors *F*_off_ and *N*_2_ using energetic arguments (Supplementary Note 1), which leads to the lower bound *F*_off_ ≃0.04–2 pN and *N*_2_≳1–50 motors. On the other hand, surprisingly no helical tube retractions were observed. This fact may be a signature of the long tails in the velocity–force curves as shown in Fig. 4a, and consequently an indirect evidence that typically *φ*<1.

## Discussion

We have shown that single-headed KIF1A motors are capable to collectively extract tubes from GUVs, under similar conditions to previous experiments with conventional kinesin. Hence, our experimental observations validate the predictions of refs 26, 27 on their high cooperativity. Our *in silico* model for longitudinal tube pulling shows a very good agreement with experiments, providing additional insight on how motors distribute at the tip and all along the tube. While for conventional kinesin the leading cluster was estimated *in silico* to involve ≲10 motors^47^, here we estimate that ≃15 KIF1A motors would be sufficient for tubulation under similar conditions. This is a remarkable result since the stall force of single-headed KIF1A is 60 times smaller than that of conventional kinesin. It is worth remarking that the choice of a saw-tooth potential to describe the KIF1A–MT interaction is only phenomenological and convenient but not essential. As previously shown in ref. 26, cooperative effects naturally arise provided that force transmission between adjacent motors is included.

A second and unexpected result is that KIF1A monomers naturally form helical nanotubes. This entails an impressive capability to exert significant off-axis forces to the point of coiling membrane tubes around MTs. To our knowledge, this is the first time that membrane tube winding around MTs is observed. We have shown that a simple mean-field model for KIF1A motors captures the essential off-axis dynamics both at a single-motor level and at a collective level. The average single-motor pitch is determined by the MT–motor interactions and the geometry of the MT lattice. The average tube pitch; however, is a collective effect resulting from the competition between the longitudinal and transversal forces generated by the motors.

In this work we have restricted ourselves to the monomeric form of KIF1A, as the simplest case study, but at the same time, the most inefficient form. We would expect dimeric KIF1A to enable a trade-off between cooperative force generation and high speed *in vivo*, due to the combination of diffusive and processive motion. This would naturally enable a motor in the processive state to push forward a diffusing motor in the front, leading to enhanced cooperativity.

Altogether, our results suggest that the existence of a diffusive state is a key distinctive feature that makes KIF1A motors genuinely cooperative for membrane-bound cargo transport and could explain their specificity to axonal vesicular traffic. Accordingly, this state affords two complementary strategies to overcome obstructions: brute force and manoeuvreing capability. In a series configuration (in line) it enables the generation of large forces by accumulation of motors, a possibility not available for conventional kinesin^26^,^27^,^29^,^50^; in a parallel configuration (side by side) it enables lateral displacement of the cargo. Further work is required to elucidate to what extent these features might be exploited by KIF1A *in vivo*.

## Methods

### KIF1A purification and labelling

A construct containing the first 382 residues of KIF1A with a His-tag and a Cys residue in the N-terminal, was kindly provided by N. Hirokawa (University of Tokyo, Japan; see ref. 13). The plasmid was expressed in *Escherichia coli* and the protein was further purified on a Ni-nitrilotriacetic acid (Ni-NTA) column. Finally, biotin labelling was done before elution. The details of the transformation, preculture and purification steps are described as follows:

The construct was transformed into *E. coli* strain BL21(DE3) (Novagen, USA). Transformed cells were grown on LB agar plates (10 g l^−1^ Tryptone, 5 g l^−1^ Yeast extract, 10 g l^−1^ NaCl and 15 g l^−1^ agar) in the presence of ampicillin (100 μg ml^−1^) and were stored at 4 °C. From a single colony, a 100 ml preculture was grown overnight at 37°C in LB medium (10 g l^−1^ Tryptone, 5 g l^−1^ Yeast extract and 10 g l^−1^ NaCl) supplemented with ampicillin (100 μg ml^−1^). The preculture was transferred to 2 l LB medium with 100 μg ml^−1^ ampicillin and further incubated at 37 °C. At *A*=0.3, cells were induced with 300 μM IPTG (isopropyl-β-D-thiogalactopyranoside) for 4 h at 30 °C. The cell culture was spun down at 5,000 r.p.m. for 30 min at 4 °C, resuspended in Lysis buffer (pH 8.0: Imidazole 20 mM, MgCl_2_ 1 mM, NaPi pH 7.0 50 mM, NaCl 250 mM, Glycerol 10%, Triton-X 0.1%, β-mercaptoethanol 5 mM, supplemented with one tablet of complete protease inhibitor cocktail per 50 ml) and flash frozen in liquid nitrogen.

The lysate was thawed at 37 °C in a water bath then quickly placed on ice. Lysosyme (1 mg ml^−1^) and a knife tip of DNAse were added to the lysate. The cell suspension was incubated in a shaking platform at 4 °C for 20 min, then submitted again to flash freeze/thaw/20 min incubation/flash freeze/thaw as described earlier. Finally, the mix was spun down at 15,000 r.p.m. for 30 min at 4 °C. The supernatant was retrieved and the His-tagged kinesins were purified with a standard Ni-NTA affinity purification, with washing buffer (pH 7.0: Imidazole 20 mM, MgCl_2_ 1 mM, NaPi pH 7 50 mM, NaCl 250 mM, Glycerol 10%, β-mercaptoethanol 5 mM and MgATP 0.1 mM) and elution buffer (pH 7.0: Imidazole 500 mM, MgCl_2_ 1 mM, NaPi pH 7.0 50 mM, NaCl 250 mM, Glycerol 10%, β-mercaptoethanol 5 mM and MgATP 0.1 mM). For labelling, before elution, 1 mM Tris(2-carboxyethyl)phosphine hydrochloride was incubated for 30 min to reduce the cysteine residues of the protein, followed by a washing step (washing buffer) and incubation for 30 min at room temperature in the presence of 8 mM labelling molecule (EZ-Link BMCC-biotin or DylightTM 550 Sulfhydryl reactive dye, Thermo scientific).

Three different types of purification were performed: no labelling, fluorescent labelling (Sulfhydryl reactive dye; SDS gel in Supplementary Fig. 4) and labelling with biotin (BMCC-biotin). The final concentrations after elution were measured with NanoDrop 2000c (Thermo scientific). The concentrations obtained were 28 μM for the unlabelled KIF1A, 42 μM for the fluorescent KIF1A and 33 μM for the biotinylated KIF1A.

### GUV formation

1,2,-Dioleoyl-sn-glycero-3-phosphocoline (DOPC), 1, 2-dioleoyl-sn-glycero-3-phosphoethanolamine-N-(cap biotinyl) (DOPE-Bio) and 1, 2-dioleoyl-sn-glycero-3-phosphoethanolamine-N-(lissamine rhodamine B sulfonyl) (DOPE-Rh) were purchased from Avanti Polar Lipids. The lipid mixture was composed of 0.1 mol% DOPE-Rh, 0.01–1 mol% DOPE-Bio (depending on the experiment) and DOPC for the remaining fraction. Ten microlitre of lipids in 1:10 chloroform/methanol were dropped onto one of two indium tin oxidecoated glass slides. The lipids were locally spread on the glass slide and dried for ∼1 h in vacuum. A 500 μl volume chamber was made with sigillum wax (Vitrex) surrounding the dried lipid area on the bottom glass. Prior to closing, the chamber was filled with a 200 mM sucrose solution. Finally, a.c. voltage was applied to the glass plates (1 V, 10 Hz) during 4 h, with the consequent formation of GUVs.

### Microtubules

MTs were prepared from tubulin purchased from Cytoskeleton. Tubulin (10 mg ml^−1^) in MRB40 (40 mM Pipes/4 mM MgCl_2_/1 mM EGTA, pH 6.8) with 1 mM GTP was incubated for 45 min at 37 °C to polymerize. MTs were stabilized by mixing them 1:10 (vol/vol) with MRB40 containing 10 μM paclitaxel (Taxol, Cytoskeleton,USA; MRB40tax). The tubulin mixture contained 10% of fluorescent tubulin (HiLyte Fluor 488).

### *In vitro* tube extraction experiments

Two-hundred microlitre of poly(-L-lysine; Sigma-Aldrich, USA) 1:500 (vol/vol) in ethanol were dropped on top of a clean coverslip and the sample was kept in the hood until complete evaporation of the drop. A circular plastic support was placed on top of the coverslip defining a 50 μl volume chamber. MTs were dropped on the chamber and incubated for 10 min to adhere. MTs that did not stick to the surface were removed by rinsing two times with MRB40tax. Casein (Sigma-Aldrich, USA) was dropped on the surface (1 mg ml^−1^) to minimize the interaction of the GUVs with the exposed glass, incubated for 10 min, and rinsed with MRB40tax. At the same time, 5 μl mix of KIF1A and streptavidin (1:1 mol) were incubated for 5 min in a rotating wheel at room temperature. GUVs were mixed 1:1 in MRB40tax with 180 mM glucose to osmotically match the intravesicular osmolarity (Osmomat 030, Cryoscopic osmometer, Gonotec, Germany). The KIF1A–streptavidin solution was mixed with the vesicle solution (around 50 μl total volume) and was incubated 5 min more in a rotating wheel. Fourty microlitre of the vesicle solution was dropped onto the chamber. Five microlitre of MRB40tax with 180 mM glucose was dropped on top of the sample to help the vesicles to settle to the glass surface. Finally, 0.5 μl of Oxygen Scavenger (8 mM DTT/0.4 mg ml^−1^ catalase/0.8 mg ml^−1^ glucose oxidase) and 2 μl of 50 mM ATP were added before observation.

### Image acquisition and analysis

Images were acquired on a total internal reflection fluorescence microscope (TIRF; Nikon Corporation, Japan) equipped with an APO TIRF × 100 1.49 numerical aperture oil objective, a motorized stage, Perfect Focus System, a motorized TIRF illuminator (Roper Scientific, Germany) and a QuantEM:512SC EMCCD camera (Photometrics, Roper Scientific). Images of moving tubes were acquired every 2 s with a spatial resolution of 158 nm. Kymographs were built using ImageJ. The data from kymographs was exported to Matlab and a homemade programme was used to fit at every time step a sigmoidal function along the nanotube to the logarithm of the intensity profile. The position of the tube tip was determined as the inflection point of the sigmoidal function, with fitting error corresponding to 1.96 s.d. (95% confidence interval). The instantaneous velocity distribution in Fig. 1g was obtained by analysing each trajectory and subtracting end point positions of a time window moving along the trace. The optimal time window size was 16 s, obtained by analysing a standing tube. For the case of helical tube pulling, the average pitch was evaluated by analysing 57 standing helical tubes. The resulting value was 1.4±0.1 μm where the error corresponds to s.e.m.

### Gliding assays

The motility of KIF1A was tested using *in vitro* gliding assays with 1:10 (vol/vol) dilutions of the purified motor solution in MRB40tax (pH 6.8: 40 mM PIPES, 4 mM MgCl_2_, 1 mM EGTA and 10 μM taxol). Motors were unspecifically attached to the glass surface in the case of unlabelled and fluorescently labelled KIF1A, and specifically attached to the glass surface via a streptavidin–biotin link using poly(L-lysine)-g-poly(ethylene glycol)-biotin (PLL-PEG-biotin, SUSOS AG, Switzerland) for the case of biotinylated KIF1A. Finally the motility solution (*κ*-casein 0.6 mg ml^−1^, methylcellulose 0.1%, glucose 50 mM, ATP 2 mM, taxol 10 μM, diluted taxol stabilized microtubules in MRB40 and oxygen scavenger system) was flushed before observation. Unlabelled and fluorescently labelled KIF1A showed gliding velocities in the range of 100–200 nm s^−1^ whereas biotinylated KIF1A smoothly moved microtubules at ≃80 nm s^−1^.

## Additional information

**How to cite this article**: Oriola, D. *et al.* Formation of helical membrane tubes around microtubules by single-headed kinesin KIF1A. *Nat. Commun.* 6:8025 doi: 10.1038/ncomms9025 (2015).

## Supplementary Material

Supplementary Information Supplementary Figures 1-4, Supplementary Table 1, Supplementary Notes 1-3 and Supplementary References

Supplementary Movie 1 Helical tube formation by single-headed kinesin KIF1A. Sequence of TIRF images of lipid fluorescence taken every 2 s during helical tube growth (x 10 speed). The movie corresponds to Fig. 1d (right).

## References

[b1] Alberts B. *et al.* Molecular Biology of the Cell Garland (2002).

[b2] Howard J. Mechanics of Motor Proteins and the Cytoskeleton Sinauer (2001).

[b3] Bálint Š., Verdeny-Vilanova I., Sandoval-Álvarez Á. & Lakadamyali M. Correlative live-cell and superresolution microscopy reveals cargo transport dynamics at microtubule intersections. Proc. Natl Acad. Sci. USA 110, 3375–3380 (2013). 23401534 10.1073/pnas.1219206110 PMC3587250

[b4] Holzbaur E. L. & Goldman Y. E. Coordination of molecular motors: from in vitro assays to intracellular dynamics. Curr. Opin. Cell. Biol. 22, 4–13 (2010). 20102789 10.1016/j.ceb.2009.12.014 PMC2846361

[b5] Hirokawa N. & Takemura R. Molecular motors and mechanisms of directional transport in neurons. Nat. Rev. Neurosci. 6, 201–214 (2005). 15711600 10.1038/nrn1624

[b6] Hirokawa N., Niwa S. & Tanaka Y. Molecular motors in neurons: transport mechanisms and roles in brain function, development, and disease. Neuron 68, 610–638 (2010). 21092854 10.1016/j.neuron.2010.09.039

[b7] Millecamps S. & Julien J.-P. Axonal transport deficits and neurodegenerative diseases. Nat. Rev. Neurosci. 14, 161–176 (2013). 23361386 10.1038/nrn3380

[b8] Okada Y., Yamazaki H., Sekine-Aizawa Y. & Hirokawa N. The neuron-specific kinesin superfamily protein KIF1A is a unique monomeric motor for anterograde axonal transport of synaptic vesicle precursors. Cell 81, 769–780 (1995). 7539720 10.1016/0092-8674(95)90538-3

[b9] Erlich Y. *et al.* Exome sequencing and disease-network analysis of a single family implicate a mutation in KIF1A in hereditary spastic paraparesis. Genome Res. 21, 658–664 (2011). 21487076 10.1101/gr.117143.110 PMC3083082

[b10] Kramer T. *et al.* Kinesin-3 mediates axonal sorting and directional transport of alphaherpesvirus particles in neurons. Cell Host Microbe 12, 806–814 (2012). 23245325 10.1016/j.chom.2012.10.013 PMC3527838

[b11] Okada Y. & Hirokawa N. Mechanism of the single-headed processivity: diffusional anchoring between the K-loop of kinesin and the C terminus of tubulin. Proc. Natl Acad. Sci. USA 97, 640–645 (2000). 10639132 10.1073/pnas.97.2.640 PMC15383

[b12] Kikkawa M. *et al.* Switch-based mechanism of kinesin motors. Nature 411, 439–445 (2001). 11373668 10.1038/35078000

[b13] Okada Y., Higuchi H. & Hirokawa N. Processivity of the single-headed kinesin KIF1A through biased binding to tubulin. Nature 424, 574–577 (2003). 12891363 10.1038/nature01804

[b14] Tomishige M., Klopfenstein D. R. & Vale R. D. Conversion of Unc104/KIF1A kinesin into a processive motor after dimerization. Science 297, 2263–2267 (2002). 12351789 10.1126/science.1073386

[b15] Hammond J. W. *et al.* Mammalian kinesin-3 motors are dimeric in vivo and move by processive motility upon release of autoinhibition. PLoS Biol. 7, e72 (2009). 19338388 10.1371/journal.pbio.1000072 PMC2661964

[b16] Soppina V. *et al.* Dimerization of mammalian kinesin-3 motors results in superprocessive motion. Proc. Natl Acad. Sci. USA 111, 5562–5567 (2014). 24706892 10.1073/pnas.1400759111 PMC3992690

[b17] Chowdhury D. Stochastic mechano-chemical kinetics of molecular motors: a multidisciplinary enterprise from a physicist's perspective. Phys. Rep. 529, 1–197 (2013).

[b18] Kolomeisky A. B. Motor proteins and molecular motors: how to operate machines at the nanoscale. J. Phys. Condens. Matter 25, 463101 (2013). 24100357 10.1088/0953-8984/25/46/463101 PMC3858839

[b19] Reimann P. Brownian motors: noisy transport far from the equilibrium. Phys. Rep. 361, 57–265 (2002).

[b20] Jülicher F., Adjari A. & Prost J. Modeling molecular motors. Rev. Mod. Phys. 69, 1269–1282 (1997).

[b21] Yildiz A., Tomishige M., Vale R. D. & Selvin P. R. Kinesin walks hand-over-hand. Science 303, 676–678 (2004). 14684828 10.1126/science.1093753

[b22] Klumpp S., Keller C., Berger F. & Lipowsky R. Multiscale Modeling in Biomechanics and Mechanobiology. Molecular motors: Cooperative phenomena of multiple molecular motors 27–61Springer (2015).

[b23] Lipowsky R., Beeg J., Dimova R., Klumpp S. & Müller M. JI. Cooperative behaviour of molecular motors: cargo transport and traffic phenomena. Physica E 42, 649–661 (2010).

[b24] Brugués J. & Casademunt J. Self-organization and cooperativity of weakly coupled molecular motors under unequal loading. Phys. Rev. Lett. 102, 118104 (2009). 19392242 10.1103/PhysRevLett.102.118104

[b25] Orlandi J. G., Blanch-Mercader C., Brugués J. & Casademunt J. Cooperativity of self-organized Brownian motors pulling on soft cargoes. Phys. Rev. E 82, 061903 (2010). 10.1103/PhysRevE.82.061903 21230686

[b26] Oriola D. & Casademunt J. Cooperative force generation of KIF1A Brownian motors. Phys. Rev. Lett. 111, 048103 (2013). 23931411 10.1103/PhysRevLett.111.048103

[b27] Oriola D. & Casademunt J. Cooperative action of KIF1A Brownian motors with finite dwell time. Phys. Rev. E 89, 032722 (2014). 10.1103/PhysRevE.89.032722 24730889

[b28] Campàs O., Kafri Y., Zeldovich K. B., Casademunt J. & Joanny J.-F. Collective dynamics of interacting molecular motors. Phys. Rev. Lett. 97, 038101 (2006). 16907545 10.1103/PhysRevLett.97.038101

[b29] Rai A. K., Rai A., Ramaiya A. J., Jha R. & Mallik R. Molecular adaptations allow dynein to generate large collective forces inside cells. Cell 152, 172–182 (2013). 23332753 10.1016/j.cell.2012.11.044

[b30] Roux A. *et al.* A minimal system allowing tubulation with molecular motors pulling on giant liposomes. Proc. Natl Acad. Sci. USA 99, 5394–5399 (2002). 11959994 10.1073/pnas.082107299 PMC122780

[b31] Koster G., VanDuijn M., Hofs B. & Dogterom M. Membrane tube formation from giant vesicles by dynamic association of motor proteins. Proc. Natl Acad. Sci. USA 100, 15583–15588 (2003). 14663143 10.1073/pnas.2531786100 PMC307611

[b32] Leduc C. *et al.* Cooperative extraction of membrane nanotubes by molecular motors. Proc. Natl Acad. Sci. USA 101, 17096–17101 (2004). 15569933 10.1073/pnas.0406598101 PMC535380

[b33] Shaklee P. M. *et al.* Bidirectional membrane tube dynamics driven by nonprocessive motors. Proc. Natl. Acad. Sci. USA 105, 7993–7997 (2008). 18332438 10.1073/pnas.0709677105 PMC2786943

[b34] Shaklee P. M., Bourel-Bonnet L., Dogterom M. & Schmidt T. Nonprocessive motor dynamics at the microtubule membrane tube interface. Biophys. J. 98, 93–100 (2010). 20085722 10.1016/j.bpj.2009.09.058 PMC2800980

[b35] Yamada A. *et al.* Catch-bond behaviour facilitates membrane tubulation by non-processive myosin 1b. Nat. Commun. 5, 3624 (2014). 24709651 10.1038/ncomms4624

[b36] Waterman-Storer C. M. & Salmon E. D. Endoplasmic reticulum membrane tubules are distributed by microtubules in living cells using three distinct mechanisms. Curr. Biol. 8, 798–806 (1998). 9663388 10.1016/s0960-9822(98)70321-5

[b37] Delevoye C. *et al.* Recyling endosome tubule morphogenesis from sorting endosomes requires the kinesin motor KIF13A. Cell. Rep. 6, 445–454 (2014). 24462287 10.1016/j.celrep.2014.01.002 PMC3928541

[b38] Skjeldal F. M. *et al.* The fusion of early endosomes induces molecular-motor-driven tubule formation and fission. J. Cell Sci. 125, 1910–1919 (2012). 22357949 10.1242/jcs.092569

[b39] Berliner E., Young E. C., Andreson K., Mahtani H. K. & Gelles J. Failure of a single-headed kinesin to track parallel to microtubule protofilaments. Nature 373, 718–721 (1995). 7854458 10.1038/373718a0

[b40] Yajima J. & Cross R. A. A torque component in the kinesin-1 power stroke. Nat. Chem. Biol. 1, 338–341 (2005). 16408073 10.1038/nchembio740

[b41] Brunnbauer M. *et al.* Torque generation of kinesin motors is governed by the stability of the neck domain. Mol. Cell. 46, 147–158 (2012). 22541555 10.1016/j.molcel.2012.04.005

[b42] Hoeprich G. J., Thompson A. R., McVicker D. P., Hancock W. O. & Berger C. L. Kinesin's neck-linker determines its ability to navigate obstacles on the microtubule surface. Biophys. J. 106, 1691–1700 (2014). 24739168 10.1016/j.bpj.2014.02.034 PMC4008791

[b43] Bormuth V. *et al.* The highly processive kinesin-8, Kip3, switches microtubule protofilaments with a bias toward the left. Biophys. J. 103, L4–L6 (2012). 22828351 10.1016/j.bpj.2012.05.024 PMC3388217

[b44] Derényi I., Jülicher F. & Prost J. Formation and interaction of membrane tubes. Phys. Rev. Lett. 88, 238101 (2002). 12059401 10.1103/PhysRevLett.88.238101

[b45] Ray S., Meyhöfer E., Milligan R. A. & Howard J. Kinesin follows the microtubule's protofilament axis. J. Cell Biol. 121, 1083–1093 (1993). 8099076 10.1083/jcb.121.5.1083 PMC2119687

[b46] Amos L. A. & Schlieper D. Microtubules and MAPs. Adv. Protein Chem. 71, 257–298 (2005). 16230114 10.1016/S0065-3233(04)71007-4

[b47] Campàs O. *et al.* Coordination of kinesin motors pulling on fluid membranes. Biophys. J. 94, 5009–5017 (2008). 18310242 10.1529/biophysj.107.118554 PMC2397365

[b48] Chowdhury D., Garai A. & Wang J.-S. Traffic of single-headed motor proteins KIF1A: effects of lane changing. Phys. Rev. E 77, 050902(R) (2008). 10.1103/PhysRevE.77.050902 18643016

[b49] Chrétien D. & Wade R. H. New data on the microtubule surface lattice. Biol. Cell. 71, 161–174 (1991). 1912942 10.1016/0248-4900(91)90062-r

[b50] Furuta K. *et al.* Measuring collective transport by defined numbers of processive and nonprocessive kinesin motors. Proc. Natl Acad. Sci. USA 110, 501–506 (2013). 23267076 10.1073/pnas.1201390110 PMC3545764

