# Author Correction: Formation of helical membrane tubes around microtubules by single-headed kinesin KIF1A

**DOI:** 10.1038/s41467-019-11010-5

**Published:** 2019-07-02

**Authors:** David Oriola, Sophie Roth, Marileen Dogterom, Jaume Casademunt

**Affiliations:** 1 Departament d’Estructura i Constituents de la Matèria, Facultat de Física, Universitat de Barcelona, Avinguda Diagonal 647, E-08028 Barcelona, Spain; 2 FOM Institute AMOLF, Science Park 104, 1098 XG Amsterdam, The Netherlands; 3 Present Address: Department of Bionanoscience, Kavli Institute of Nanoscience, TU Delft, Lorentzweg 1, 2628 CJ Delft, The Netherlands

Correction to: *Nature Communications* 10.1038/ncomms9025, published online 13 August 2018.

This Article contains an error in the first sentence of the ‘KIF1A purification and labelling’ section of the Methods, which incorrectly reads ‘A construct containing the first 382 residues of KIF1A with a His-tag and a Cys residue in the N-terminal, was kindly provided by N. Hirokawa (University of Tokyo, Japan; see ref. ^13^).’ This should say ‘C-terminus’ instead of ‘N-terminus’. The error has not been fixed in the PDF or HTML versions of the Article.

