# Supplementary material for: Formation of helical membrane tubes around microtubules by single-headed kinesin KIF1A
Source: Nat Commun. 2015 Aug 13;6:8025. doi: 10.1038/ncomms9025 (PMC4557341; doi:10.1038/ncomms9025)
Supplement: Supplementary Information — Supplementary Figures 1-4, Supplementary Table 1, Supplementary Notes 1-3 and Supplementary References [file ncomms9025-s1.pdf]

Supplementary Figures

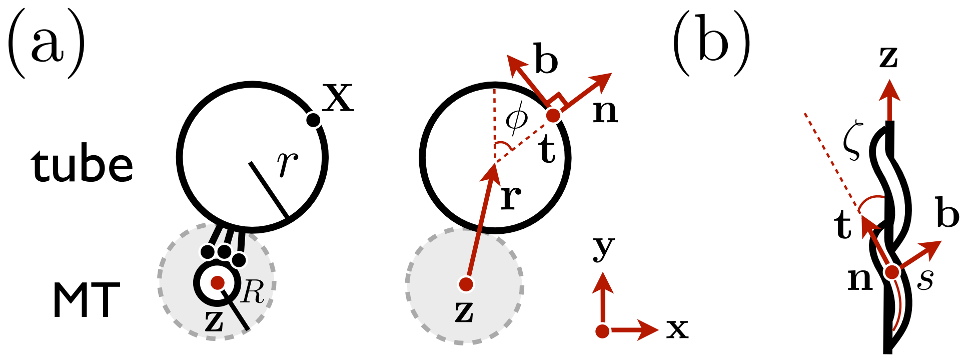

Figure 1: Geometry of a tubular helix winding around a MT. a) Sketch of the tube with radius r and an imaginary cylinder of radius R depicted as a dashed circle containing the grey region. The radius of this cylinder corresponds to the radius of the MT plus some extra space occupied by the motors. We associate an orthonormal triad $\{\mathbf{t}, \mathbf{n}, \mathbf{b}\}$ at each point $\mathbf{X}(s, \phi)$ on the surface of the helical tube. The vector $\mathbf{r}(s)$ defines the position of the nanotube center at s, and the angle ϕ defines a point on the tube surface. b) Helical tube forming a helical angle ζ with respect to the MT axis.

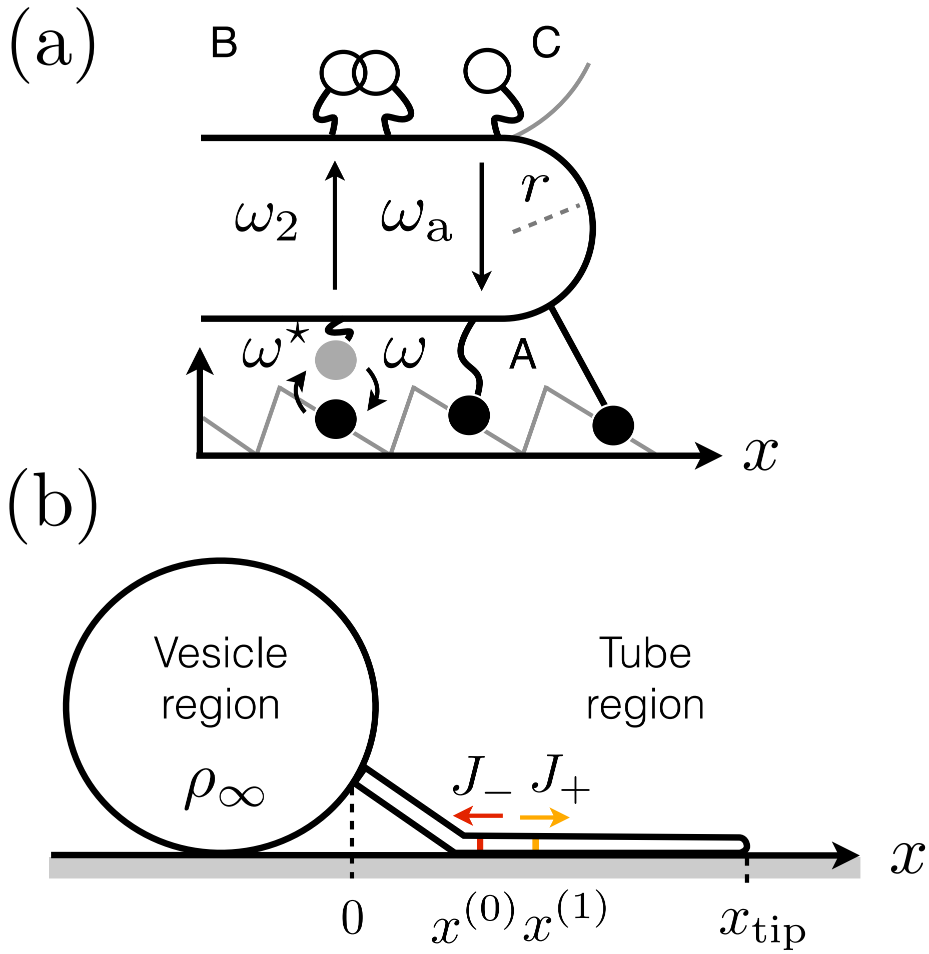

Figure 2: Schematic description of the *in silico* model. a) Three different regions can be identified in the tube region (A, B and C). In region A, motors are either strongly bound (state 1, black circles) or weakly bound (state 2, grey circles) to the MT. They are excited and decay with average rates $\omega^\star, \omega$ respectively. In region B, motors are detached from the MT (state 0, white circles) and diffuse freely outside the curved region of size r in the tip. Overlapping is allowed to account for the two-dimensional diffusive motion of motors on the membrane. Motors detach from state 2 and attach from state 0 with mean rates ω_2, ω_a respectively. Finally, in region C, detached motors feel a soft repulsive potential V which prevents them to enter the curved region. b) Tube extracted from a GUV with surface density of motors ρ_∞. An influx J_+ of motors enters the tube region at $x^{(1)}$ and an outflux J_- enters back to the GUV at $x^{(0)}$.

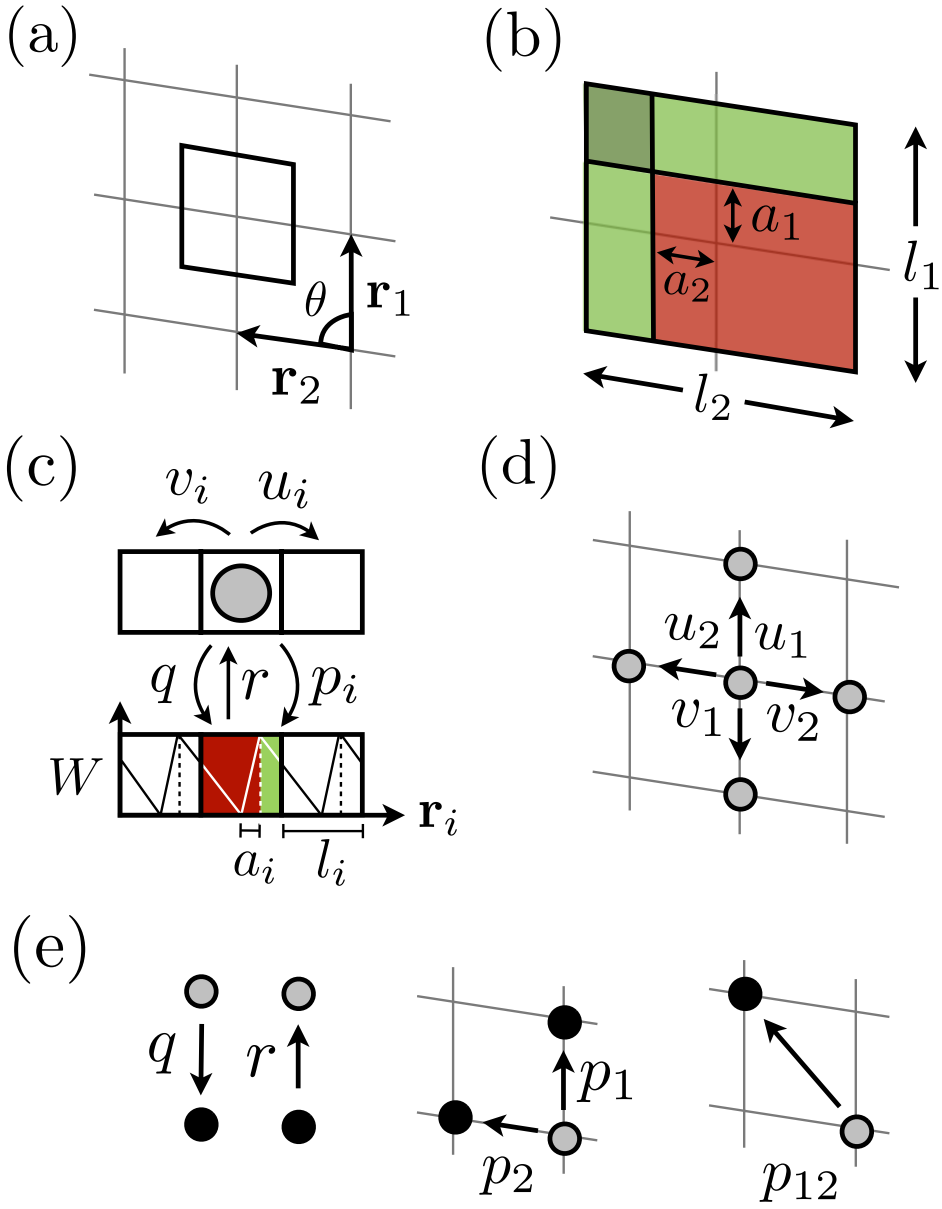

Figure 3: Microscopic model for single-headed KIF1A in a 2D lattice. a) Two-dimensional oblique Bravais lattice representing the MT surface with directions $\mathbf{r}_1$, $\mathbf{r}_2$, forming an angle θ. The lattice unit is centered in each node. b) Lattice unit with lengths l_1 and l_2 and ratchet asymmetries a_1, a_2. c) Schematic description of a two-state noise-driven ratchet for the i-th direction and its equivalent description on a lattice. The transition p_{12} is not depicted in this case. Motors can diffuse with rates u_i, v_i and advance with rates p_i along the i-th direction. d) Two-dimensional diffusion on the lattice in the weakly bound state. e) Possible transitions between the strongly bound state (black circles) and the weakly bound state (grey circles) and its corresponding transition rates.

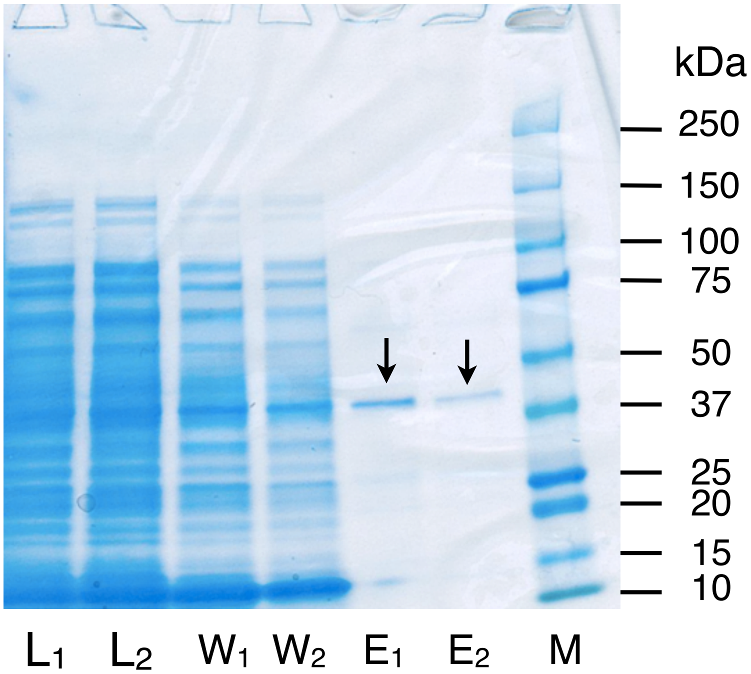

Figure 4: SDS gel for fluorescently labeled (1) and unlabeled (2) KIF1A. From left to right: protein lysate (L), first wash (W), elution (E) and Kaleidoscope marker (M). The two arrows correspond to the $\simeq$ 45 kDa band, corresponding to the KIF1A monomer molecular weight.

Supplementary Table 1

Table 1: Parameters used in the *in silico* model

Parameter	Value
MT periodicity length	$l = 8$ nm
Motor size	$\sigma = 4.2$ nm
Ratchet asymmetry	$a = 1.8$ nm
Excitation window	$\delta = 0.16$ nm
Characteristic detachment length	$d = 3$ nm
Ratchet potential maximum	$U = 10\, k_{\mathrm{B}}T$
Surface tension	$\gamma = 0.05$ pN nm^{-1}
Bending rigidity	$\kappa = 10\, k_{\mathrm{B}}T$
GUV surface motor density	$\rho_\infty = 200 - 1000$ μm^{-2}
Diffusion coefficient (MT)	$D = 20$ nm^2 ms^{-1}
Diffusion coefficient (tube)	$D_{\mathrm{t}} = 1$ μm^2 s^{-1}
Excitation rate	$\omega^\star = 33$ s^{-1}
Decay rate	$\omega = 250$ s^{-1}
Detachment rate (zero load)	$\omega_2^0 = 1$ s^{-1}
Attachment rate	$\omega_{\mathrm{a}} = 3$ s^{-1}

Supplementary Note 1: Geometry and energetics of a tubular helix

We consider a tubular helix of radius r (tube radius) winding on an imaginary cylinder of radius R (MT radius plus some extra space occupied by the motors, depicted as a dashed circle in Supplementary Fig. 1a, left), such that $r+R$ is the distance between the center of the MT and the center of the tube. The helix moves along the z-axis with an angular pitch p which is the distance the helix advances per radian along the z-axis. Each point on the surface of the helix is determined by the vector $\mathbf{X}$. We can parametrize every point on the helix surface with two variables $\{s, \phi\}$, where s is the arclength following the path of the helix through its center and ϕ is an angle which determines at a given arclength s the position of a point in the circle of radius r. If we call a the angle the helix moves per arclength unit, the distance the helix advances along the z-axis after moving a distance s along the helix will be $p\theta$, where $\theta \equiv as$. If we move an arclength unit, we are moving a distance ap in the z-axis and a distance aR_0 in the axis perpendicular to the z-axis, where $R_0 \equiv R + r$. In this way we find $1 = a^2(R_0^2 + p^2)$. On the other hand, the angle ζ the center of the helix forms respect to the z-axis fulfills (see Supplementary Fig. 1b):

$$\tan\zeta = \frac{R_0}{p} \tag{S1}$$

The position of the center of the left-handed helix is $\mathbf{r}(s) = (R_0 \sin\theta, R_0 \cos\theta, p\theta)$. We can parametrize the surface of the tubular helix $\mathbf{X}(s, \phi)$ as:

$$\mathbf{X}(s, \phi) = \mathbf{r}(s) + r[\mathbf{n}(s)\cos\phi + \mathbf{b}(s)\sin\phi] \tag{S2}$$

with $\phi \in [0, 2\pi)$, using the orthonormal triad $\{\mathbf{t}, \mathbf{n}, \mathbf{b}\}$ such that $\mathbf{t}(s) = \frac{\partial_s \mathbf{r}}{|\partial_s \mathbf{r}|}$, $\mathbf{n}(s) = \frac{\partial_s \mathbf{t}}{|\partial_s \mathbf{t}|}$ and $\mathbf{b}(s) = \frac{\mathbf{t}\wedge\mathbf{n}}{|\mathbf{t}\wedge\mathbf{n}|}$ (see Supplementary Fig. 1a, right). The mean curvature of the surface $H(\phi)$ can be determined through the first and second fundamental forms $g_{\mu\nu}(s, \phi) = \partial_\mu \mathbf{X} \cdot \partial_\nu \mathbf{X}$ and $\Pi_{\mu\nu}(s, \phi) = \partial_{\mu\nu}\mathbf{X} \cdot \mathbf{n}$, $\mu, \nu = s, \phi$; which in a matrix form read:

$$\mathbf{g} = \begin{pmatrix} E & F \\ F & G \end{pmatrix} \quad \mathbf{\Pi} = \begin{pmatrix} L & M \\ M & N \end{pmatrix} \tag{S3}$$

The mean curvature can be calculated through the expression $H(\phi) = \frac{EN - 2FM + GL}{2\det(\mathbf{g})}$ [1, 2] which leads to:

$$H(\phi) = \frac{1}{2}\left[\frac{1}{r} + rK(\phi)\right] \tag{S4}$$

where $K(\phi) = -\frac{C\cos\phi}{r(1 - rC\cos\phi)}$ and $C = \frac{R_0}{R_0^2 + p^2}$ is the curvature of the helical spine curve of the tube. We notice that for $p \to \infty$ the curvature tends to the one of a cylinder of radius r i.e. $1/2r$. The total surface of a tubular helix of lenght L and radius r is $2\pi rL$, the same as for a cylinder. Consequently, the surface energy is $2\pi rL\gamma$, and it does not depend on the pitch. Let us define the small quantity $\epsilon \equiv rC$. This quantity is bounded below 1 in the experiments. Considering the first correction up to fourth order in ϵ, the free energy of the system can be approximated as:

$$\mathcal{F} \simeq \frac{\pi\kappa L}{r}\left(1 + \frac{1}{2}\epsilon^2\right) + 2\pi rL\gamma - FL \tag{S5}$$

In the limit $p \to \infty$ (or $\epsilon \to 0$), we recover the free energy of a cylindrical tube with extraction force $F = 2\pi\sqrt{2\kappa\gamma}$ and diameter $2r = \sqrt{\kappa/\gamma}$. Therefore, we conclude that the last two expressions for the cylindrical case are accurate to second order in ϵ and yield good approximations to the actual values for the helical tubes.

Superhelical effect on the tube pitch

Let us consider a helical tube of pitch P growing around a MT which has superhelicity of pitch P_{sh}. The angle that the tube will advance per arclength unit will be:

$$a(p') = a(p) + a(p_{\mathrm{sh}}) = a(p)(1+\xi) \tag{S6}$$

where p' is the angular pitch, p is the angular pitch for a 13pf MT and ξ is the relative increase in angle which reads:

$$\xi = \sqrt{\frac{1+\cot^2\zeta}{1+\cot^2\zeta_{\mathrm{sh}}}} \tag{S7}$$

where $\cot\zeta_{\mathrm{sh}} = p_{\mathrm{sh}}/R_0$.

Estimation of the off-axis force exerted by the motors

Next we estimate the off-axis force exerted by the motors by using free energy arguments. From Eq. S5 we find that a lower bound of the excess free energy $\Delta\mathcal{F}$ associated to the winding of the tube reads:

$$\Delta\mathcal{F} \simeq \frac{\pi\kappa L}{2r}\epsilon^2 \tag{S8}$$

This excess free energy is due to the work KIF1A motors perform in the off-axis direction W_{off} along the tube. This work can be estimated to be:

$$W_{\mathrm{off}} = 2\pi R_{\mathrm{MT}} N_{\mathrm{w}} F_{\mathrm{off}} \tag{S9}$$

where F_{off} is the total off-axis force exerted by the motors and N_{w} is the winding number. Actually, the above expression underestimates the work by taking the radius of the displacement as that of the microtubule, and not that of the point at the membrane where the force is exerted. The winding number can be expressed in terms of the angle the helix moves per arclength unit a as $N_{\mathrm{w}} = La/2\pi$. Equating the last two expressions we obtain the total off-axis force:

$$F_{\mathrm{off}} = \frac{\pi\kappa\epsilon^{3/2}}{2R_{\mathrm{MT}}}\sqrt{1+\frac{R}{r}} \tag{S10}$$

In our experiments $\epsilon \simeq 0.04 - 0.5$. Which gives a lower bound of the total off-axis force in the range $F_{\mathrm{off}} \simeq 0.04 - 2$ pN.

Supplementary Note 2: In silico model for longitudinal tube pulling

We illustrate the problem of tube-pulling for a tube of radius r and extraction force F, considering N motors in the vesicle reservoir and assuming that motors use a single pf [1] extending the model presented in Ref. [3]. We distinguish two main regions in the system: the tube region and the vesicle region (Supplementary Fig. 2b).

Tube region

A subset of motors $N_\mathrm{t} \subset N$ are found in the tube region at time t. A motor i from this subset is found in state $k_i(t)$ at time t, where k_i is a discrete stochastic variable. Motors can be detached from the MT ($k_i = 0$), strongly bound to the MT ($k_i = 1$) or weakly bound to the MT ($k_i = 2$). In all cases, motors are also bound to the tube considered as a soft cargo. The dynamics of each motor is different depending on the region where it is found (A, B or C, see Supplementary Fig. 2a). Next we describe the dynamics in each region:

Region A corresponds to the region in between the tube and the MT, were motors can be either weakly or strongly bound to the MT (i.e. $k_i \neq 0$). In this region the dynamics reads [3]:

$$\lambda \dot{x}_i = -U'(x_i, k_i) - \sum_{j \in \mathcal{S}} W'(x_i - x_j) - F\delta_{x_i, x_{\max}} + \zeta_i^{(D)}(t) \qquad \text{(S11)}$$

where λ is the friction coefficient, such that the diffusion coefficient follows the Einstein relation $D = k_\mathrm{B}T/\lambda$. $U(x_i, k_i)$ is the potential motors feel depending on the state k_i. In the strongly bound state ($k_i = 1$), motors feel a periodic saw-tooth potential of asymmetry a and periodicity l. The results are not significantly affected by the particular form of the ratchet potential as shown in previous studies where similar results are obtained in a lattice model [3, 4]. On the other hand, in the weakly bound state ($k_i = 2$) they feel a constant potential $U(x_i, 2) = U_2$. W accounts for the motor-motor interaction potential and is taken as a truncated Lennard-Jones potential of hard-core size σ and energy minimum ϵ, where the former corresponds to the motor size and the latter is taken large enough to ensure that the interaction is effectively hardcore for motor-motor distances smaller than σ. The i-th motor interacts only with a subset of motors $\mathcal{S}(t)$ at time t which are bound to the MT, i.e. $\mathcal{S}(t) = \{j | j \neq i, k_j(t) \neq 0\}$. $\zeta^{(D)}(t)$ is a Gaussian white noise with $\langle \zeta_i^{(D)}(t) \zeta_j^{(D)}(t') \rangle = 2k_\mathrm{B}T\lambda\delta_{ij}\delta(t-t')$. Finally $x_{\max}$ corresponds to the position of the foremost motor in the system.

In region B, motors are detached from the MT ($k_i = 0$) and undergo free diffusion on the membrane tube outside the curved region in the tip of size r. Defining $\xi_i = x_i - x_\mathrm{tip}$ as the relative distance between the position of the i-th motor and the tip position x_tip, the condition for a detached motor to be in region B is $|\xi_i| > r$. The dynamics reads:

$$\lambda_\mathrm{t} \dot{\xi}_i = \zeta_i^{(D_\mathrm{t})}(t) \qquad \text{(S12)}$$

where λ_t, D_t are the friction and diffusion coefficients on the membrane tube respectively. We notice there is no interaction potential for the motors in this region since we allow overlapping to account for the two-dimensional diffusive motion of motors on the tube. In this way, motors are no longer ordered respect to their label i.

Region C corresponds to the curved region of the tip where $|\xi_i| < r$. In this case we neglect noise and the dynamics simply read:

$$\lambda_\mathrm{t} \dot{\xi}_i = -V'(\xi_i) \qquad \text{(S13)}$$

[1] The case of N_p pf is done by simply scaling the extraction force F and the surface density of motors ρ_∞ by N_p.

with a repulsive potential V in the form of a truncated Morse potential which is valid for $|\xi_i| < r$ and is zero otherwise. This is an *ad hoc* choice to simply prevent motors to enter in region C. We adjust the Morse parameters to ensure a soft short-ranged repulsive potential preventing the detached motors to enter the curved region. We associate the dynamics of the tube tip with the dynamics of the foremost motor i.e. $\dot{x}_{\mathrm{tip}} = \dot{x}_{\mathrm{max}}$. If the foremost motor detaches, the tube retracts with a retraction velocity $\dot{x}_{\mathrm{tip}} = -F/\lambda_{\mathrm{m}}$ until a new bound motor is found. λ_{m} is an effective friction parameter which is inferred from the retraction velocity of motors $v_{\mathrm{r}} \simeq 100$ μm s^{-1} observed experimentally for $F \simeq 20$ pN [5]. Finally we estimate the number of bound motors in the tip over time by counting the number of consecutive bound motors pulling on the tube, where we define two motors as consecutive if they are at a distance less than $\delta_D = \sqrt{4D/\omega}$. Also, we estimate the density of bound motors from the set of positions of the bound motors at each time step of the simulation using a smoothing technique. We define the density of motors at each point as the number of bound motors in a characteristic bandwidth divided by its length. The bandwidth size is taken $\simeq 100$ nm, which is similar to the experimental spatial resolution.

Next we describe the state dynamics. All state transitions in the system are stochastic with dwell times which are exponentially distributed. Motors are excited from state 1 to 2 from localized regions of size δ near the minima of the ratchet potential with average rate $\omega^{\star}$. On the other hand, decays from state 2 to 1 are delocalized with average rate ω. Attachment events occur with average rate ω_{a}. However, they are not always possible due to excluded volume interactions in region A. Thus, we say that a motor i will only attach if it can find a free site i.e. $\sum_{j\in\mathcal{S}} W'(x_i - x_j) = 0$. We include exponentially dependent detachment kinetics on the force. *In vitro* experiments using single-headed kinesin have shown that the detachment rate at zero load is much larger in the weakly bound state ($\sim$ 1 s^{-1}) than in the strongly bound state (~ 0.01 s^{-1}) [6]. We choose to neglect detachment from state 1 for simplicity. The addition of detachment in state 1 leads to similar dynamics in the system. The average detachment rate from state 2 of the i-th motor at time t, will depend on the passive forces the i-th motor feels over time:

$$\omega_2(i,t) = \omega_2^0 \exp\left(\frac{|\tilde{F}_i(t)|d}{k_{\mathrm{B}}T}\right) \tag{S14}$$

where ω_2^0 is the detachment rate at zero load from state 2, $\tilde{F}_i(t)$ is local time-averaging of the noisy signal of the passive forces $F_i(t) = -\sum_{j\in\mathcal{S}} W'(x_i - x_j) - F\delta_{x_i,x_{\mathrm{max}}}$ and d is a characteristic length which is typically 2 to 4 nm for kinesin [6, 7, 8]. We used a simple smoothing technique by choosing $\tilde{F}_i(t)$ as the time average of F_i in the region $[t-\tau, t]$, where τ is the window size. This is taken as $\tau = 100$ ms, which is big enough to average the passive forces a motor feels during a hydrolysis cycle ($\sim$ 10 ms), and smaller than the timescale of the tube motion ($\sim$ 1 s). Variations of τ around this value did not affect significantly the dynamics of the system.

Vesicle region

The vesicle region is described as a motor reservoir with surface density of motors ρ_∞. Motors diffuse on the vesicle and eventually they enter the tube region through the boundary $x = x^{(0)}$ (Supplementary Fig. 2b, red line). Hence, in the boundary we have an influx of motors $J_+(t)$ which are bound to the MT and to the tube. Experimental evidences show that it is feasible to neglect the influx of motors only bound to the tube [9]. On the other hand, there is also a flux of motors leaving the tube $J_-(t)$ by diffusion. Since KIF1A is able to make large backward excursions in the weakly bound state, for practical reasons it is important to ensure motors will not fluctuate near the boundary $x = x^{(0)}$. Hence, we let motors appear at $x^{(1)} = x^{(0)} + \delta_D$, (Supplementary Fig. 2b, orange line) and let $x = x^{(0)}$ act as an absorbing boundary condition for the motors that leave the tube region. The number of motors in this region $N_{\mathrm{t}}(t)$ will depend on time through the flux balance:

$$\frac{dN_{\mathrm{t}}}{dt} = J_+ - J_- \tag{S15}$$

Experimental evidences indicate that Eq. (S15) reaches a quasi-steady state [9]. Far from the tip, in the quasi-steady state the density of motors bound to the tube in the mean-field limit reads [5]:

$$\rho_{\rm b} = 2\pi r \rho_\infty \frac{\omega_{\rm a}}{\omega_{\rm d}^0 + \omega_{\rm a}} \tag{S16}$$

where in our case $\omega_{\rm d}^0 = \omega_2^0/(1+\beta)$ is the motor detachment rate at zero load and $\beta = \omega/\omega^\star$. Therefore, at the boundary $x^{(1)}$, the average influx of motors will be $\langle J_+ \rangle = \rho_{\rm b} V_0$. In our simulations, a new motor will be introduced in the system stochastically every certain time taken from an exponential distribution with mean dwell time $\langle J_+ \rangle^{-1}$. Finally, motors crossing the boundary $x^{(0)}$ will be incorporated in the vesicle reservoir.

Discussion on realistic parameters for the in silico model

The most determinant characteristic of single-headed KIF1A is that its movement relies on diffusion. *In vitro* experiments have reported diffusion coefficients in the range of 20 to 40 nm^2 ms^{-1} which involve motor excursions much larger than the ratchet periodicity of 8 nm [10] . We will consider 20 nm^2 ms^{-1} as a reasonable value. The asymmetry a of the ratchet is an adjustable parameter for the model which is difficult to grasp from experiments. The asymmetry reduces the overall velocity of the system and it can lead to non-trivial effects specially in the limit of weak noise [11]. For our purposes, we adjust this parameter to 20 % of the periodicity length. Finally, the motor size σ is carefully chosen to avoid possible commensurability effects [11, 12]. The characteristic rates ω and $\omega^\star$ are found in the literature within the range of hundreds of Hz. Whereas ω is a parameter coming from the affinity between the motor domain and the MT, $\omega^\star$ depends on ATP concentration in the solvent. Experimental data suggest that $\omega^\star \leq 250$ s^{-1} and $\omega \simeq 250$ s^{-1} [10, 13]. We choose β such that the velocity of a single KIF1A at zero load is similar to the experimental gliding velocities ~ 80 nm s^{-1} (see Methods). The resulting value is $\beta \simeq 7.5$. The detachment rate of KIF1A has been found to be $\omega_{\rm d} \sim 0.1$ s^{-1}. Using our value of β we get $\omega_2^0 \simeq 1$ s^{-1}, which is in agreement with the results in Ref. [6].

The radius of the tube r and the threshold force to extract a tube F depend on the bending rigidity κ and the surface tension of the membrane γ through the expressions $r = \sqrt{\kappa/(2\gamma)}$ and $F = 2\pi\sqrt{2\kappa\gamma}$ [14]. κ is assumed to be roughly constant in experiments whereas γ can vary substantially. Although in principle the surface tension can be adjusted *in vitro* by changing the osmotic pressure difference in the system, the statistical dispersion of γ from vesicle to vesicle is large and makes it difficult to control this parameter. The typical range of γ implicitly obtained through our data analysis is $3 \times 10^{-4} - 10^{-1}$ pN nm^{-1}. The density of motors in the vesicle can be obtained assuming that each lipid occupies a surface of approximately 0.4 nm^2. In the experiments we used different fractions of biotinylated lipids, in the range 0.01-1 mol %. This range corresponds to $\simeq 200 - 20000$ μm^{-2}. The diffusion coefficient on the tube is much larger than the diffusion coefficient in the weakly bound state ($D_{\rm t} \gg D$). Typically, $D_{\rm t} \simeq 1\,\mu$m^2 s^{-1} [9]. Finally, the allowed range of attachment rates is reported to be 0.1 s^{-1} $\leq \omega_{\rm a} \leq 10$ s^{-1} [13], and we will take an intermediate value. The Supplementary Table 1 shows a summary of the selected values for the different parameters.

Supplementary Note 3: Lattice model for a single KIF1A motor

Here we present a phenomenological approach to describe the motion of a single-headed KIF1A motor moving in a two-dimensional lattice under an external load by taking into account the presence of two asymmetry parameters. In order to describe the motion of a single KIF1A motor on a 2D MT lattice, we extend the lattice description in Ref. [3] by considering a 2D oblique Bravais lattice (Supplementary Fig. 3a) with directions $\mathbf{r}_1$ and $\mathbf{r}_2$ forming an angle θ. The vector $\mathbf{r}_1$ describes the on-axis motion of kinesin reflecting the polarity of the MT. On the other hand, the vector $\mathbf{r}_2$ shows the off-axis motion determined by the bias due to the interaction of the motor domain and the MT lattice. The mechanism of motion can be understood by considering a properly generalized two-state model in two dimensions.

We define the state of the motor as $k = 1, 2$ depending on whether the motor is strongly bound ($k = 1$) or weakly bound ($k = 2$) to the MT. In the strongly bound state, we consider the motor feels a superposition of two asymmetric ratchet potentials forming an angle θ with asymmetries a_i and periodicities l_i (Supplementary Fig. 3b, c) where $i = 1, 2$ indicate the directions in the lattice. Each node in the lattice indicates the landscape minima. A motor in the strongly bound state can be excited with rate $r = \omega^\star$ to the weakly bound state in which it undergoes two-dimensional diffusion on the lattice with rates u_i, v_i, $i = 1, 2$ (Supplementary Fig. 3d). These rates depend linearly on an external force $\mathbf{F} = (F_1, F_2)$ as $u_i = (d_i/2)(1 - f_i)$, $v_i = (d_i/2)(1 + f_i)$, $i = 1, 2$, where $f_i = F_i l_i / k_\mathrm{B}T$ is the dimensionless force, $d_i = D/l_i^2$ is the diffusion rate and D is the one-dimensional diffusion coefficient in the weakly bound state. When the motor decays, it can fall in one of the four possible regions depicted in Supplementary Fig. 3b. If the motor falls in the red region, it performs a q transition binding strongly to the MT in the same node (Supplementary Figs. 3c and 3e.). However, if the motor falls in one of the three green regions, it will move to a new node in the lattice. The motor will make a p_1 transition if it falls in the upper green region, a p_2 transition if it falls in the left green region, and a p_{12} transition if it falls in the dark green region. The latter probabilities are equal to the decay rate ω times the probability of falling in a given region, which can be directly obtained calculating the areas in Supplementary Fig. 3b. We define a given transition rate from state $\{k, \mathbf{R}\}$ to $\{k', \mathbf{R}'\}$ as $\Gamma(k', \mathbf{R}'|k, \mathbf{R})$. The different transition rates read:

$$\begin{aligned}
\Gamma(1, \mathbf{R} + \mathbf{r}_1 + \mathbf{r}_2|2, \mathbf{R}) &= p_{12} = \frac{\omega}{4}(1 - 2\bar{a}_1)(1 - 2\bar{a}_2) \\
\Gamma(1, \mathbf{R} + \mathbf{r}_1|2, \mathbf{R}) &= p_1 = \frac{\omega}{4}(1 - 2\bar{a}_1)(1 + 2\bar{a}_2) \\
\Gamma(1, \mathbf{R} + \mathbf{r}_2|2, \mathbf{R}) &= p_2 = \frac{\omega}{4}(1 + 2\bar{a}_1)(1 - 2\bar{a}_2) \\
\Gamma(1, \mathbf{R}|2, \mathbf{R}) &= q = \frac{\omega}{4}(1 + 2\bar{a}_1)(1 + 2\bar{a}_2) \\
\Gamma(2, \mathbf{R}|1, \mathbf{R}) &= r = \omega^\star \\
\Gamma(2, \mathbf{R} + \mathbf{r}_i|2, \mathbf{R}) &= u_i = \frac{d_i}{2}(1 - f_i) \\
\Gamma(2, \mathbf{R} - \mathbf{r}_i|2, \mathbf{R}) &= v_i = \frac{d_i}{2}(1 + f_i)
\end{aligned} \tag{S17}$$

where $\bar{a}_i \equiv a_i/l_i$. The mean velocities in the $\mathbf{r}_1$ and $\mathbf{r}_2$ directions will be:

$$\begin{aligned}
v_1 &= l_1 \sigma_2^\mathrm{ss}(p_{12} + p_1 + u_1 - v_1) \\
v_2 &= l_2 \sigma_2^\mathrm{ss}(p_{12} + p_2 + u_2 - v_2)
\end{aligned} \tag{S18}$$

where $\sigma_2^\mathrm{ss} \equiv 1/(1 + \beta)$ is the steady state probability of the motor to be found in state $k = 2$ and $\beta \equiv \omega/\omega^\star$. Substituting the form of the different rates, we can write the last expressions as:

$$v_i = \frac{v_i^\mathrm{d}}{1 + \beta}(f_i^\mathrm{s} - f_i); \quad i = 1, 2 \tag{S19}$$

where $v_i^{\rm d} \equiv l_i d_i$ and $f_i^{\rm s} = \frac{\omega}{2d_i}(1-2\bar{a}_i)$ is the dimensionless stall force in the direction $\mathbf{r}_i$. We notice we recover the results for the one-dimensional case in each direction [3]. The last expression can be rewritten in terms of the velocity at zero load $v_i(0)$ and stall force $F_i^{\rm s}$ as:

$$v_i(F_i) = v_i(0)\left(1 - \frac{F_i}{F_i^{\rm s}}\right), \quad i = 1, 2 \tag{S20}$$

where $v_i(0) = (l_i\omega/2)(1-2\bar{a}_i)/(1+\beta)$ and $F_i^{\rm s} = (k_{\rm B}T\omega/l_i d_i)(1-2\bar{a}_i)$. Using the KIF1A parameters from the Supplementary Table 1, we get $v_1(0) \simeq 70$ nm s^{-1} and $F_1^{\rm s} \simeq 0.1$ pN.

Supplementary References

[1] Kamien, R.D. The geometry of soft materials: a primer. *Rev. Mod. Phys.* **74,** 953-971, (2002).

[2] Gray, A. *Modern Differential Geometry of Curves and Surfaces.* (CRC Press, Boca Raton, 1993).

[3] Oriola, D. & Casademunt, J. Cooperative force generation of KIF1A Brownian motors. *Phys. Rev. Lett.* **111,** 048103, (2013).

[4] Oriola, D. & Casademunt, J. Cooperative action of KIF1A Brownian motors with finite dwell time. *Phys. Rev. E* **89,** 032722, (2014).

[5] Campàs, O. *et al.* Coordination of kinesin motors pulling on fluid membranes. *Biophys. J.* **94,** 5009-5017, (2008).

[6] Uemura, S. *et al.* Kinesin-microtubule binding depends on both nucleotide state and loading direction. *Proc. Natl. Acad. Sci. USA.* **99,** 5977-5981, (2002).

[7] Schnitzer, M. J., Visscher, K. & Block, S.M. Force production by single kinesin motors. *Nat. Cell. Biol.* **2,** 718-723, (2000).

[8] Schroeder III, H.W., *et al.* Force-dependent detachment of kinesin-2 biases track switching at cytoskeletal filament intersections. *Biophys. J.* **103,** 48-58, (2012).

[9] Leduc, C. *et al.* Cooperative extraction of membrane nanotubes by molecular motors. *Proc. Natl. Acad. Sci. USA.* **101,** 17096-17101 (2004).

[10] Okada, Y., Higuchi, H. & Hirokawa, N. Processivity of the single-headed kinesin KIF1A through biased binding to tubulin. *Nature*, **424,** 574-577, (2003).

[11] Orlandi, J. G., Blanch-Mercader, C., Brugués, J. & Casademunt, J. Cooperativity of self-organized Brownian motors pulling on soft cargoes. *Phys. Rev. E* **82,** 061903, (2010).

[12] Brugués, J. & Casademunt, J. Self-organization and cooperativity of weakly coupled molecular motors under unequal loading. *Phys. Rev. Lett.* **102,** 118104, (2009).

[13] Nishinari, K., Okada, Y., Schadschneider, A. & Chowdhury, D. Intracellular transport of single-headed molecular motors KIF1A. *Phys. Rev. Lett.* **95,** 118101, (2005).

[14] Derényi, I., Jülicher, F. & Prost, J. Formation and interaction of membrane tubes. *Phys. Rev. Lett.* **88**, 238101, (2002).
